# Single-nucleus genomics in outbred rats with divergent cocaine addiction-like behaviors reveals changes in amygdala GABAergic inhibition

**DOI:** 10.1038/s41593-023-01452-y

**Published:** 2023-10-05

**Authors:** Jessica L. Zhou, Giordano de Guglielmo, Aaron J. Ho, Marsida Kallupi, Narayan Pokhrel, Hai-Ri Li, Apurva S. Chitre, Daniel Munro, Pejman Mohammadi, Lieselot L. G. Carrette, Olivier George, Abraham A. Palmer, Graham McVicker, Francesca Telese

**Affiliations:** 1 Bioinformatics and Systems Biology Program, University of California San Diego, La Jolla, CA USA; 2 Integrative Biology Laboratory, Salk Institute for Biological Studies, La Jolla, CA USA; 3 Department of Psychiatry, University of California, San Diego, La Jolla, CA USA; 4 Department of Medicine, University of California San Diego, La Jolla, CA USA; 5 Department of Integrative Structural and Computational Biology, The Scripps Research Institute, La Jolla, CA USA; 6 Center for Immunity and Immunotherapies, Seattle Children’s Research Institute, Seattle, WA USA; 7 Department of Pediatrics, University of Washington School of Medicine, Seattle, WA USA; 8 Department of Genome Sciences, University of Washington, Seattle, WA USA; 9 Institute for Genomic Medicine, University of California San Diego, La Jolla, CA USA

**Keywords:** Epigenetics and behaviour, Reward, Addiction, Bioinformatics, RNA sequencing

## Abstract

The amygdala processes positive and negative valence and contributes to addiction, but the cell-type-specific gene regulatory programs involved are unknown. We generated an atlas of single-nucleus gene expression and chromatin accessibility in the amygdala of outbred rats with high and low cocaine addiction-like behaviors following prolonged abstinence. Differentially expressed genes between the high and low groups were enriched for energy metabolism across cell types. Rats with high addiction index (AI) showed increased relapse-like behaviors and GABAergic transmission in the amygdala. Both phenotypes were reversed by pharmacological inhibition of the glyoxalase 1 enzyme, which metabolizes methylglyoxal—a GABA_A_ receptor agonist produced by glycolysis. Differences in chromatin accessibility between high and low AI rats implicated pioneer transcription factors in the basic helix-loop-helix, FOX, SOX and activator protein 1 families. We observed opposite regulation of chromatin accessibility across many cell types. Most notably, excitatory neurons had greater accessibility in high AI rats and inhibitory neurons had greater accessibility in low AI rats.

## Abstract (web summary)

Using single-cell genomics, addiction-like behaviors in outbred rats are associated with persistent cell-type-specific molecular signatures in the amygdala, implicating a pivotal role of energy metabolism in cocaine addiction.

## Main

The amygdala regulates numerous behaviors related to emotions, motivation and memory^1^ and is implicated in various neuropsychiatric disorders including addiction^2,3^. Repeated drug use engages the amygdala to form drug-associated memories and reinforces drug-seeking behavior^4^. In addition, during withdrawal from addictive drugs, the amygdala mediates negative emotional states, such as anxiety, fear and irritability^4^. Avoidance of these aversive emotions enhances the incentive value of the drug, leading to sustained drug-seeking behaviors and relapse^5–7^. The amygdala is composed of several interconnected subregions^8^ including the basolateral amygdala (BLA) and the central amygdala (CeA)^9,10^. While the behavioral function and connectivity of the amygdala have been established^1^, the role of distinct neuronal and non-neuronal cell subpopulations in addiction remains unclear.

Recently developed single-nucleus RNA-sequencing (snRNA-seq) and single-nucleus assays for transposase-accessible chromatin (snATAC-seq) have enabled the study of the cellular function and diversity of the human, mouse and nonhuman primate brains^11–17^. However, their application to study the neurobiology of addiction has been limited. snRNA-seq has been applied to characterize cellular diversity in brain regions involved in the reward system^18–21^ and has been used to analyze transcriptional changes induced by experimenter-administered cocaine and morphine in rodents^22,23^. However, these previous studies used inbred rodent strains, which limited examination of genetically mediated differences in susceptibility to addiction-like behaviors. Furthermore, these studies focused on acute drug treatments and therefore did not explore molecular changes that accompany long-lasting addictive-like behaviors.

To address these limitations, we performed snRNA-seq and snATAC-seq using amygdala tissue from outbred heterogenous stock (HS) rats obtained from a large genetic study of cocaine addiction-related traits^24^. These rats were exposed to extended access drug intravenous self-administration (IVSA)^24–26^. IVSA is linked to neurochemical changes in key brain regions, such as those observed in humans with cocaine use disorder^27^. HS rats were used because they have high levels of genetic variation and rich phenotypic diversity^28–31^.

## Results

### Outbred rats exhibit low or high cocaine addiction-like traits

**Fig. 1:**
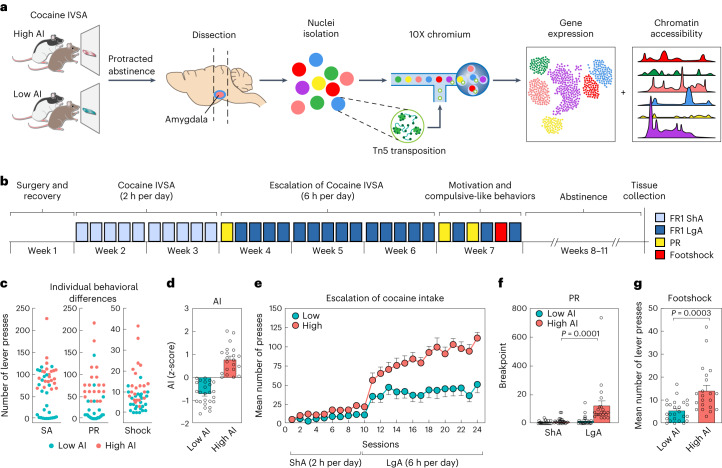
Experimental design and rat IVSA cocaine model of addiction. **a**, Schematic of the study design. **b**, Timeline of the behavioral protocol. **c**, Individual differences in total number of cocaine rewards in self-administration (SA), PR and shock-paired (Shock) sessions for each rat. **d**, Mean AI scores in high and low AI rats. **e**, Mean number of cocaine rewards across each ShA and LgA IVSA session in high (*n* = 21) and low (*n* = 25) AI rats. **f**, Breakpoint analysis of high (*n* = 21) and low (*n* = 25) AI rats under ShA versus LgA (unpaired two-sided Studentʼs *t*-test with Bonferroni adjusted *P* = 0.0001; ShA versus LgA for high AI rats, *t*_41_ = 4.525). **g**, Mean number of cocaine rewards when paired with electric footshock in high (*n* = 21) and low AI (*n* = 25) rats (*P* = 0.0003; unpaired two-sided Studentʼs *t*-test, *t*_44_ = 3.936). Error bars in **d**–**g** represent s.e.m.

To study the impact of cocaine on cellular states associated with addiction-like behaviors, we performed snRNA-seq and snATAC-seq on amygdala tissues from HS rats following 4 weeks of abstinence from cocaine IVSA^24,32–35^ (Fig. 1a). The animals were trained to self-administer cocaine in operant chambers via lever press (fixed ratio of 1 with 0.5 mg kg^–1^ per infusion) for 10 short access (ShA, 2 h per day, 5 days per week) followed by 14 long access (LgA, 6 h per day, 5 days per week) sessions. We measured the number of cocaine rewards, or lever presses, during each session of the behavioral protocol. Escalation of intake was determined as the increase in the mean number of cocaine rewards during LgA sessions compared with the first day of the LgA phase. Motivation for cocaine was assessed at the end of ShA and LgA phases, using a progressive ratio (PR) schedule of reinforcement, where the number of lever presses required to obtain a cocaine infusion increased progressively. Compulsive-like behavior was measured as drug taking despite adverse consequences by pairing 30% of lever presses with an electric footshock (Fig. 1b). For each rat (Fig. 1c), we calculated an AI^24^ as the average of the normalized values (*z*-scores) of these three behavioral measures.

We classified rats into high and low AI groups (Fig. 1d). Both groups received fewer cocaine rewards in ShA compared with LgA sessions (two-way repeated-measures analysis of variance (ANOVA), AI × phase interaction *P* < 0.0001, *F*_23,1012_ = 8.523; Fig. 1e). The groups showed no difference in cocaine rewards during ShA sessions; however, a contrasting pattern in escalation emerged during LgA sessions, where rats with high AI exhibited a progressive escalation of drug intake compared with rats with low AI (two-way repeated-measures ANOVA interaction time × group *F*_13,572_ = 4.175, *P* < 0.0001; Fig. 1e). In contrast, low AI rats did not show escalation during the LgA sessions (Fig. 1e). During PR sessions, motivation for cocaine increased in high AI rats but not in low Al rats when comparing ShA versus LgA (mixed effect model, AI × phase interaction, *P* = 0.0049, *F*_1,41_ = 8.83; Bonferroni corrected *P* = 0.0001, post hoc comparisons; Fig. 1f). Finally, high AI rats received a higher number of cocaine infusions when the reward was paired with an electric footshock (*P* < 0.001, unpaired two-sided Student’s *t*-test, *t*_44_ = 3.936; Fig. 1g), indicating compulsive-like drug use. These results show that our model of extended access to cocaine IVSA in outbred rats captures several relevant aspects of cocaine use disorder.

### snRNA-seq and snATAC-seq define cell types in the amygdala

To identify neuroadaptations that persist in the amygdala after chronic drug exposure during withdrawal, we measured the gene expression and chromatin accessibility profiles of individual nuclei by performing snRNA-seq and snATAC-seq after 4 weeks of abstinence (Fig. 1a). We performed snRNA-seq on 19 rats, including 6 with high AI, 6 with low AI and 7 naive rats never exposed to cocaine (Supplementary Data 1); and snATAC-seq on 12 rats, including 4 with high AI, 4 with low AI and 4 naive rats (Supplementary Data 2).

We obtained a combined total of 163,003 and 81,912 high quality nuclei from the snRNA-seq and snATAC-seq samples, respectively (Supplementary Figs. 1–7 and Supplementary Data 3–4). Using the integrated snRNA-seq and snATAC-seq datasets, we identified 49 and 41 cell-type clusters, respectively (Supplementary Fig. 8). Visualization of the integrated data indicated that the clustering is not influenced by batch effects such as sequencing library, percentage of mitochondrial DNA or individual rats^36^ (Supplementary Fig. 9).

**Fig. 2:**
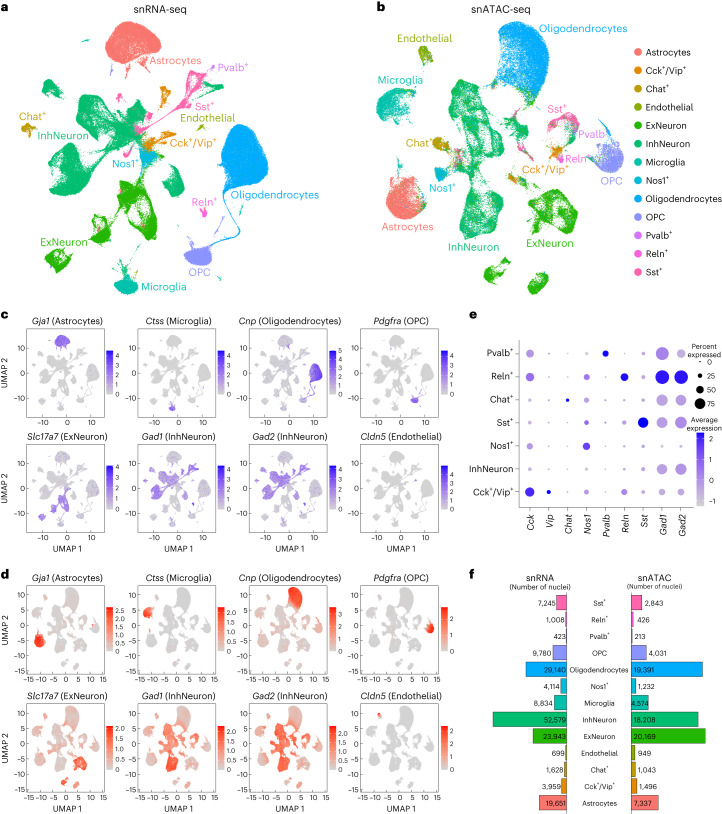
Summary of single-nucleus RNA-seq and ATAC-seq data from rat amygdala. **a**, UMAP plot of snRNA-seq data from rat amygdala. Data are combined across 19 samples, with high, low and naive AI labels. Cells are colored by cluster assignments performed with KNN analysis. We assigned cell-type labels to clusters based on the expression of known marker genes. **b**, UMAP plot of snATAC-seq data from 12 rat amygdala samples. snATAC-seq data were integrated with snRNA-seq data, and cluster labels were transferred to snATAC-seq cells. **c**, Feature plot showing expression of marker genes used to label main subsets of cells: *Gja1* (astrocytes), *Ctss* (microglia), *Cnp* (oligodendrocytes), *Pdgfra* (OPCs), *Slc17a7* (excitatory neurons), *Gad1*/*Gad2* (inhibitory neurons) and *Cldn5* (endothelial cells). **d**, Feature plot showing imputed gene expression of cell-type-specific marker genes in snATAC-seq dataset. **e**, Expression of marker genes in cell clusters corresponding to highly specific subsets of inhibitory neurons. The shading and diameter of each circle indicate the estimated mean expression and the percentage of cells in the cluster in which the marker gene was detected. **f**, The number of nuclei assigned to each cell-type cluster for the snATAC-seq and snRNA-seq datasets.

Using established cell-type-specific marker genes^11,37–40^, we annotated the snRNA-seq clusters (Fig. 2a,b) into main cell types, including excitatory neurons (*Slc17a7*), inhibitory GABAergic neurons (*Gad1/Gad2*), astrocytes (*Gja1*), microglia (*Ctss*), mature oligodendrocytes (*Cnp*), oligodendrocyte precursor cells (OPC) (*Pdgfra*) and endothelial cells (*Cldn5*) (Fig. 2c). The annotation of the snATAC-seq clusters using the imputed gene expression of cell-type markers (Supplementary Fig. 10) clearly delineated the cell clusters into the same main cell types described above, demonstrating strong concordance between our snRNA-seq and snATAC-seq data (Fig. 2d). We also identified seven subtypes of inhibitory neurons based on the expression of known cell marker genes (Fig. 2e), and subclustered the excitatory neurons to identify 18 distinct clusters (Supplementary Fig. 11), with top marker genes including known subpopulation markers such as *Cdh13*, *Nr4a2* and *Bdnf* (ref. ^41^). Cell-type proportions seemed to be consistent across samples (Supplementary Figs. 12 and 13). The total number of nuclei we obtained for each cell type varied substantially (Fig. 2f and Supplementary Table 1). For most downstream analyses, we focused on the six most common main cell types (Fig. 2a,b).

To relate the cell types in the whole amygdala to those in spatially defined amygdalar subregions, we generated snRNA-seq data from the CeA and BLA (Supplementary Fig. 14). Cell clusters from the CeA and BLA were distinct from one another, but these regions collectively contained most cell types identified in the whole amygdala (Supplementary Fig. 14a). Consistent with the known cell-type composition of the CeA and BLA^42^, cell clusters from the CeA coclustered primarily with inhibitory neurons whereas those from the BLA coclustered with excitatory neurons (Supplementary Fig. 14b). Glial cell types from the whole amygdala contained cells from both subregions, except for astrocytes, which coclustered mostly with cells from the CeA but not those from the BLA, suggesting that astrocytes might play a specific role in CeA-related function (Supplementary Fig. 14a,b).

The snRNA-seq and snATAC-seq datasets we generated are the first single-cell atlas of molecularly defined cell types in the rat amygdala under normal conditions and during cocaine addiction-like behaviors.

### Gene expression differences between high and low AI rats

**Fig. 3:**
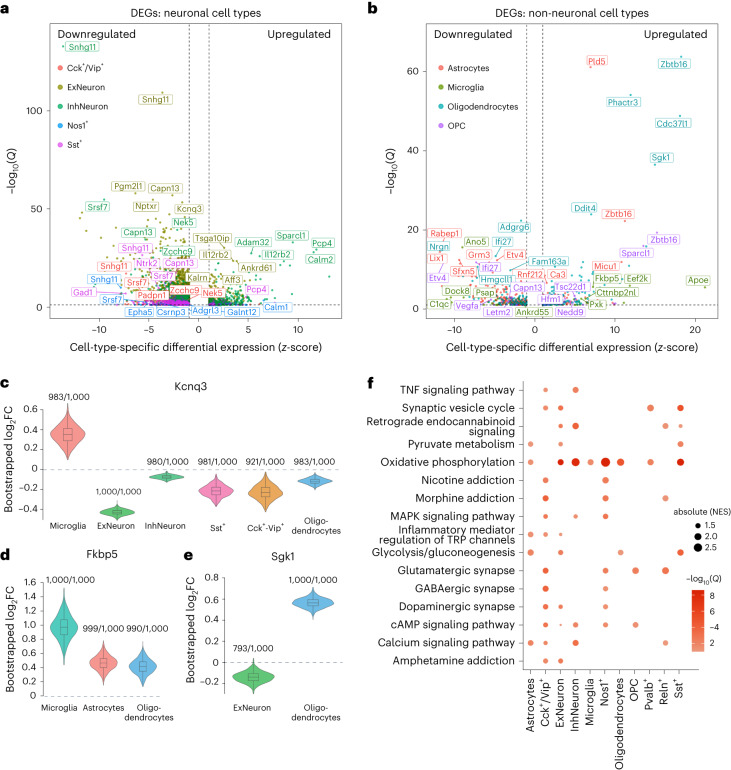
Differential gene expression between high and low AI rats. **a**, Volcano plot summarizing differential gene expression between high and low AI rats based on a two-sided negative binomial test. Points are colored by cell type, and the five most significant (FDR < 10%) up- and downregulated genes in each cell type are indicated with labels. In each cell type, we normalized the logFC values reported by Seurat to convert to *z*-scores and plotted the cell-type-specific *z*-scores on the *x* axis (*z* > 0 indicates higher expression in high AI rats; *z* < 0 indicates higher expression in low AI rats). The –log_10_FDR-corrected *P* values (*Q* values) are plotted on the *y* axis. **b**, Volcano plot summarizing differential gene expression based on a two-sided negative binomial test between high and low AI rats for non-neuronal (glial) cell-type clusters. **c**–**e**, Violin and embedded boxplots showing distribution of log_2_FC from the negative binomial (negbinom) test performed in 1,000 bootstrap iterations. Fractions indicate the number of bootstrap iterations in which the log_2_FC estimate was significantly different from 0. Boxplot hinges are the 25th and 75th percentiles; whiskers extend to the minimum and maximum; center line is the median and dotted line is the mean. Bootstrap distributions were obtained for cell types in which the following genes had significant differential expression (FDR < 10%): *Kcnq3* (**c**), *Fkbp5* (**d**) and *Sgk1* (**e**). **f**, KEGG pathways that are enriched for DEGs by cell type. Dot size indicates –log_10_(*Q*) while color indicates normalized enrichment score (NES), which is a metric of GSEA. Only pathways/cell types where *Q* < 0.1 are visualized. MAPK, mitogen-activated protein kinase; TNF, tumor necrosis factor; TRP, transient receptor potential.

We used the negative binomial test to identify differentially expressed genes (DEGs) between high and low AI rats in each cell type (Fig. 3a,b and Supplementary Data 5). To control for violations in the differential expression model (for example, overdispersion) that can cause overly significant *P* values^43,44^, we performed the same statistical test after permuting the AI labels of the rats, which removes any association between AI and gene expression. These permutation tests indicated that the highly significant DEGs were not due to poor *P* value calibration (Supplementary Fig. 15 and Supplementary Data 6).

We grouped DEGs into small (absolute(log_2_fold change (FC)) < 0.1) or large (absolute(log_2_FC) ≥ 0.1) effect size groups and observed that most significant DEGs (false discovery rate (FDR) < 10%) had small effect sizes (Supplementary Fig. 16). In total, we identified 557 unique significant DEGs with large effects in at least one cell type and 8,775 unique significant DEGs with small effects in at least one cell type. These DEG could reflect inherited differences in gene expression that predate exposure to cocaine, or they could be caused by differences in the amount of self-administered cocaine. Consistent with the former, we found that significant DEGs were enriched for gene expression quantitative trait loci (eQTLs)^45^, which are genetic variants associated with the expression of a gene, in almost every cell type tested (Chi-squared test with 1 d.f.; *P* < 0.05) (Supplementary Fig. 17 and Supplementary Table 2). Among the most significant DEGs with eQTLs (Supplementary Data 7) were genes with reported roles in substance use disorders. For example, *Kcnq3* was differentially regulated across neuronal and glial cell types, and encodes a subunit of a potassium channel implicated in the regulation of reward behavior and susceptibility to drug addiction (Fig. 3c)^46,47^. Additionally, *Fkbp5* and *Sgk1*, two transcriptional targets of the glucocorticoid receptor, were differentially regulated in glial cell types and are associated with reward behavior and drug addiction vulnerability (Fig. 3d,e)^48–50^.

To further examine the contribution of genetics to observed differences in gene expression, we leveraged genotypes and gene expression data from a reference population of drug-naive HS rats^45^. This allowed us to predict gene expression based on cis-genetic variation in the absence of cocaine exposure. Specifically, we trained models to predict gene expression from single nucleotide polymorphism genotypes^51^ using whole-brain bulk RNA-seq from 339 naive HS rats, and estimated the fraction of variance in expression that was explained by cis-genetic variation (*r*^2^). We used the trained models to predict the expression of genes with at least one cis-acting eQTL (8,997 genes) for each of the rats in our snRNA-seq dataset and compared the differences in mean predicted expression in high versus low AI rats with the observed differences in expression for each cell type after filtering out genes with low *r*^2^ (Supplementary Table 3). The observed and predicted expression differences were significantly correlated (Spearman’s *ρ*, *P* < 0.05) for microglia, oligodendrocytes and inhibitory neurons, and increasing the stringency of the *r*^2^ cutoff increased the strength of these correlations (Supplementary Fig. 18 and Supplementary Table 3). These observations indicate that genetic differences in high versus low AI rats contribute to some of the observed differences in expression. Cocaine exposure probably also plays a role; however, quantifying the relative contributions of cocaine and genetics is challenging due to limitations in the genetic predictions of gene expression.

To identify pathways with altered regulation between high and low AI rats, we performed gene set enrichment analysis (GSEA)^52^ of Kyoto Encyclopedia of Genes and Genomes (KEGG) pathways. We identified significant enrichment of several pathways related to addiction, including neurotransmission and energy metabolism (Fig. 3f and Supplementary Data 8). Most cell types showed enrichment of genes belonging to the oxidative phosphorylation pathway, which, together with glucose metabolism, is the main energy source for synaptic activity and action potentials^53,54^. These observations suggest that addiction-like behaviors are associated with alterations in the metabolic state of amygdalar cell populations, which can directly impact neural network activity in the amygdala.

### AI is linked to GABAergic transmission

**Fig. 4:**
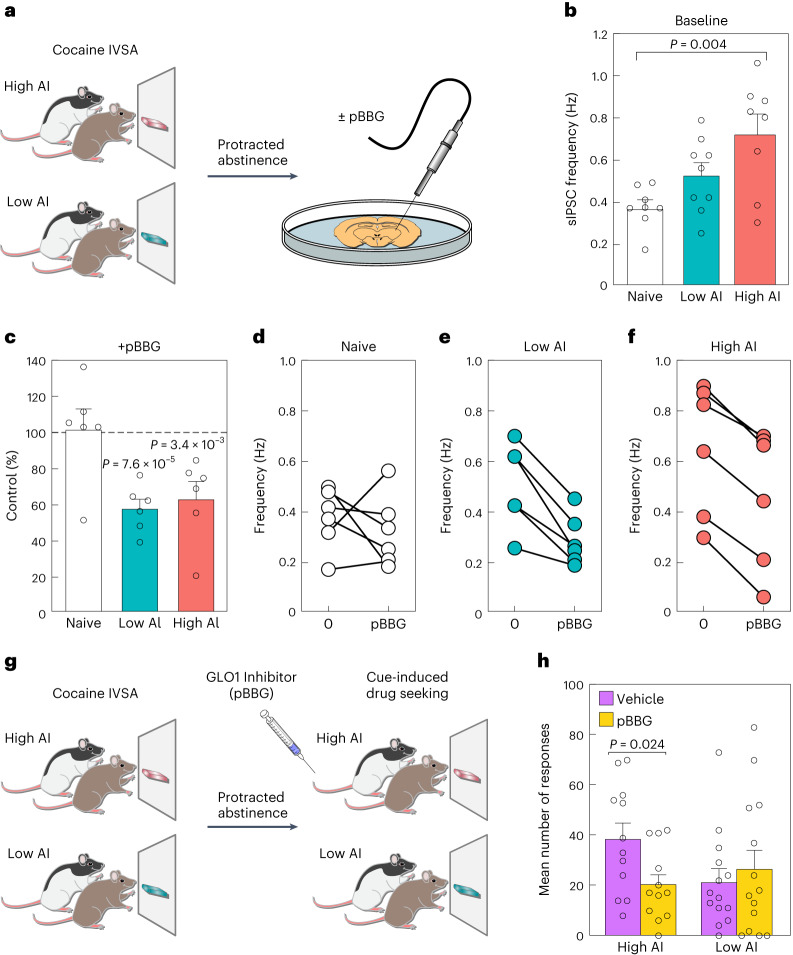
Electrophysiology and GLO1 inhibition experiments implicate GABAergic inhibition in cocaine addiction-like behaviors. **a**, Schematic showing animal model used for electrophysiology recording in CeA slices from HS rats subjected to 4 weeks of abstinence from cocaine IVSA. Electrophysiological recordings were taken before and after pBBG treatment from tissue slices of five naive, five low AI and five high AI rats. **b**, Baseline sIPSC frequency before pBBG injection. A significant difference between the means of the naive versus high AI rats was observed (adjusted *P* = 0.004, Tukey’s honestly significant difference test). **c**, sIPSC frequency following pBBG treatment. We observed significantly reduced frequency in the CeA slices from high and low AI rats but not in naive rats when we compare baseline versus pBBG in each group (*P*_high_ = 7.6 × 10^–5^; *P*_low_ = 3.4 × 10^–3^, *P*_naive_ = 0.51, paired two-sided Student’s *t*-test). **d**–**f**, Change in sIPSC frequency following pBBG treatment in naive (**d**), low AI (**e**) and high AI (**f**) rats. **g**, Schematic of animal model used to test cue-induced cocaine-seeking behavior. Rats with low and high AI were injected with vehicle or pBBG following a period of prolonged abstinence, and re-exposed to SA chambers in the absence of cocaine. **h**, Following injection of pBBG, cocaine-seeking behavior in high AI rats (*n* = 12), but not low AI rats (*n* = 14), was reduced by pBBG treatment (unpaired Student’s *t*-test with Bonferroni adjusted *P* = 0.024, vehicle versus pBBG in high AI rats). Error bars in panels **b**, **c**, and **h** represent s.e.m.

To test the hypothesis that altered cellular metabolic state impacts neural activity in the amygdala, we focused on GABAergic transmission, which has been implicated previously in addiction^2^. Specifically, we measured GABAergic transmission by recording spontaneous inhibitory postsynaptic currents (sIPSCs) in the CeA. CeA slices were collected after 4 weeks of abstinence from a separate cohort of five low AI and five high AI HS rats exposed to the same behavioral protocol described for the snRNA-seq and snATAC-seq experiments (Fig. 4a). We recorded baseline GABAergic transmission using CeA slices prepared from five age-matched naive HS rats. There were differences in mean sIPSC frequencies among the groups (one-way ANOVA *F*_2,22_ = 6.77, *P* = 0.0051), reflecting a progressive increase in GABAergic transmission from naive to low AI to high AI groups (Fig. 4b and Supplementary Fig. 19a), without detectable changes in amplitude (Supplementary Fig. 19b,c). These results support the hypothesis that the cocaine addiction-like behaviors in high AI rats reflect increased GABAergic transmission.

To further investigate the link between GABAergic transmission and energy metabolism in the amygdala with cocaine addiction-like behaviors, we measured the frequency and amplitude of sIPSCs before and after application of S-bromobenzylglutathione cyclopentyl diester (pBBG)^55,56^. pBBG is an inhibitor of glyoxalase 1 (GLO1), the rate-limiting enzyme for the metabolism of methylglyoxal (MG), which is a byproduct of glycolysis that is a competitive partial agonist of GABA_A_ receptors^55^. We found that pBBG reduced the sIPSC frequency compared with vehicle for both high and low AI rats (paired *t*-tests, *t*_5_ = 11.83, *P* = 7.6 × 10^–5^ and *t*_5_ = 5.07, p = 3.9 × 10^–3^, respectively), but not naive rats (*t*_5_ = 0.71, *P* = 0.51) (Fig. 4c–f and Supplementary Fig. 19a). We observed no effect of pBBG on sIPSCs amplitude (Supplementary Fig. 19b,c).

The above results led us to hypothesize that GLO1 inhibition might reverse behavioral differences observed following prolonged abstinence from cocaine IVSA. Thus, we measured cue-induced reinstatement of cocaine-seeking behavior in a separate cohort of 26 low and high AI rats following 4 weeks of abstinence from cocaine IVSA. Rats were injected with pBBG or vehicle 30 min before testing^57^ (Fig. 4g). Rats were subjected to the same operant conditions of cocaine IVSA but without drug availability, and reinstatement was triggered by re-exposure to the cocaine infusion-associated light cue. A significant interaction between AI and pBBG treatment (two-way repeated-measures ANOVA, *F*_1,24_ = 6.609, *P* < 0.05) indicated that pBBG reduced cue-induced reinstatement in high AI rats (*P* value < 0.05, post hoc comparisons with Bonferroni correction), but not in low AI rats (*P* > 0.05). These results demonstrate that modulating GABA_A_ transmission via the pharmacological inhibition of GLO1 decreases relapse-like behaviors in animals with high cocaine AI.

### Chromatin accessibility changes in high versus low groups

**Fig. 5:**
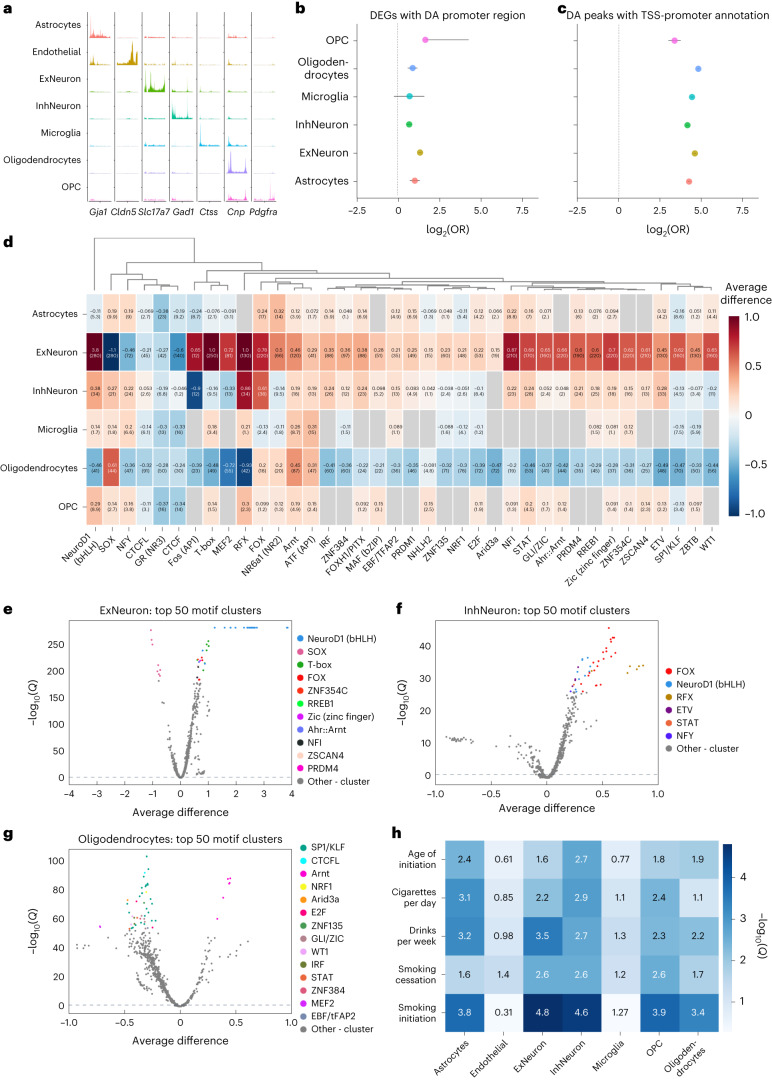
Analysis of chromatin accessibility and regulatory elements involved in cocaine dependence. **a**, Pseudobulk chromatin accessibility at the promoter regions of marker genes for main cell types. **b**, Significant DEGs (FDR < 10%) for each main cell type are enriched for promoters with DA chromatin. Points are log_2_OR (odds ratio) and error bars are 95% CIs (FDR < 10%; two-sided FET, *n* = 12,081 genes for astrocytes, *n* = 12,590 for ExNeuron, *n* = 12,679 for InhNeuron, *n* = 11,232 for microglia, *n* = 11,886 for oligodendrocytes and *n* = 11,646 for OPC). This indicates that the snRNA-seq and snATAC-seq results are consistent and that gene expression changes are associated with changes in promoter chromatin accessibility. **c**, Cell-type-specific DA peaks are enriched in TSS/promoter regions compared with non-TSS/promoter regions. Points are log_2_OR and error bars are 95% CIs (FDR < 10%; two-sided FET, *n* = 291,844 peaks) **d**, Heatmap showing differential activity of various motifs in the significant differential peaks of each cell type. Values indicate average difference of chromVAR deviation scores with –log_10_(*Q*) in parentheses, where *Q* is the Benjamini–Hochberg FDR-corrected *P* value from a two-sided Wilcoxon signed rank test for difference in deviation scores. There are many cases where motifs display increased activity in upregulated peaks in neurons while also displaying decreased activity in downregulated peaks in oligodendrocytes. **e**–**g**, Volcano plots showing average (mean) difference (*x* axis) and –log_10_(*Q*) (*y* axis) of chromVAR deviation scores for the top 50 motif clusters in excitatory neurons (**e**), inhibitory neurons (**f**) and oligodendrocytes (**g**). **h**, LD score regression results showing significance of enrichment of heritability for several traits related to alcohol and nicotine addiction in cell-type-specific accessible chromatin regions (mapped to hg19). Significance is reported as –log10(*Q*), where *Q* is the Benjamini–Hochberg FDR-corrected *P* value obtained from the ldsc software^98^.

We used MACS2 (ref. ^58^) to identify regions of accessible chromatin from the snATAC-seq data. The pseudobulk chromatin accessibility showed the expected cell-type-specific patterns at the transcription start sites (TSS) of marker genes for each cell type (Fig. 2c,d and Fig. 5a), indicating the expected relationship between chromatin accessibility and transcriptome measurements.

To better understand the regulatory mechanisms involved in cocaine addiction, we performed negative binomial^59,60^ tests to measure cell-type-specific differential chromatin accessibility between high and low AI rats (Supplementary Data 9) and compared the *P* values observed with those obtained from permuted data, which confirmed that the differential peaks between high and low AI are statistically significant (Supplementary Fig. 20 and Supplementary Data 10). In total, we identified >20,000 peaks across cell types, with significant differential accessibility between the high and low AI groups (FDR < 10%). However, most differences were small (log_2_FC < 0.1) (Supplementary Fig. 21), indicating that differences in addiction-like behaviors between rats are associated with modest regulatory changes at a large number of sites.

The differential peaks were categorized into those with higher (upregulated) or lower (downregulated) accessibility in the high AI rats (Supplementary Fig. 21). Astrocytes had roughly equal numbers of up- and downregulated peaks, but other cell types showed profound directional biases. Excitatory neurons were the most biased, with only two downregulated peaks detected and >8,000 upregulated peaks in the high AI group. Inhibitory neurons showed the opposite bias, with >4,000 downregulated peaks but only ~500 upregulated peaks in the high AI group (Supplementary Fig. 21). These biases probably reflect altered activity of transcription factors (TFs) controlling large transcriptional programs.

To determine whether the differential chromatin accessibility is consistent with the differential gene expression, we overlapped the significant differentially accessible (DA) chromatin peaks in each cell type with the promoters of significant DEGs and observed a significant enrichment (Fisher’s exact test (FET), *P* < 0.05) at the promoters of DEGs compared with non-DEGs (Fig. 5b and Supplementary Table 4), including the promoter regions for genes belonging to the oxidative phosphorylation pathway in inhibitory neurons, excitatory neurons and oligodendrocytes (Supplementary Fig. 22 and Supplementary Table 5). These findings confirm that the differences in chromatin accessibility and gene expression are concordant.

In total, 3.2% of the significant differential peaks were annotated as promoter or TSS regions (Supplementary Fig. 23 and Supplementary Dataset 11), which is a substantial enrichment given the genomic annotations of all accessible chromatin regions in the main cell types (FET, FDR < 10%) (Fig. 5c and Supplementary Table 6). This enrichment may indicate that changes in chromatin associated with addiction-like behaviors are more concentrated at promoters, or that we have greater statistical power to detect changes at promoters, due to larger effect sizes or greater chromatin accessibility.

We hypothesized that differences in chromatin accessibility between high and low AI rats are caused by differential TF activity. To test this hypothesis, we analyzed the snATAC-seq data using ChromVAR (Supplementary Dataset 12), which identifies TF motifs associated with differential accessibility using sparse single-cell data^61^. As many TFs recognize similar motifs, we grouped them into motif clusters and summarized results across cell types (Fig. 5d).

The motif cluster with the most significant difference in accessibility between high and low AI rats contained motifs for basic helix-loop-helix (bHLH) TFs. This motif cluster had substantially higher accessibility in the excitatory neurons of high AI rats compared with low AI rats (deviance 3.8; *P* = 1 × 10^–280^), and a modest increase in accessibility in inhibitory neurons (deviance 0.38; *P* = 1 × 10^–34^) (Fig. 5e–g). The top-ranked motifs in this cluster all harbored the sequence CAGATGG, which closely matches binding site motifs for several neuronal pioneer TFs, including those of the bHLH, RFX and FOX families^62,63^. Thus, the widespread increases in chromatin accessibility in excitatory neurons of high AI rats could reflect increased activity of pioneer TFs that recruit chromatin remodelers. However, we did not observe corresponding upregulation in the expression of genes encoding TFs belonging to these clusters (Supplementary Data 5 and Supplementary Data 12), suggesting that a different mechanism might affect their activity.

Many motif clusters with increased accessibility in the neurons of high AI rats have decreased accessibility in oligodendrocytes (Fig. 5d–g). Prominent among these motif clusters are those containing FOX and RFX motifs (Fig. 5d–g). Several motif clusters also have opposite effects between excitatory and inhibitory neurons, including SOX, MEF2 and Fos (activator protein 1 (AP1)) motifs. AP1 and MEF2 motifs are implicated in addiction^64–67^ and their expression changes in the brain following chronic exposure to cocaine and other drugs^68–72^. Consistent with these results, we observed decreased expression of genes encoding AP1 TFs including *Fosl1*, *Fos*, *Jun*, *Junb* and *Jund* in inhibitory neurons of high AI rats compared with low AI rats (Supplementary Fig. 24), suggesting that differences in their expression level affect their regulatory activity. These results implicate many motif clusters associated with addiction-like behaviors across thousands of regulatory regions and in a cell-type-specific manner.

To assess whether our rat snATAC-seq data is relevant for human addiction-related traits, we mapped the accessible chromatin peaks to the human reference genome and performed cell-type-specific linkage disequilibrium (LD) score regression^73^ using summary statistics from well-powered genome-wide association studies (GWAS) for alcohol and tobacco use^74,75^. We found significant enrichments (FDR < 10%) of single nucleotide polymorphism heritability in every trait tested in almost every cell type (Fig. 5h), with the most significant enrichments in neurons, astrocytes, oligodendrocytes and OPCs. These results indicate that the regulatory architecture of HS rats is relevant for human addiction-related traits.

## Discussion

To better understand the molecular basis of addiction, we generated an atlas of single-cell gene expression and chromatin accessibility in the amygdala of rats with divergent cocaine addiction-like behaviors after a prolonged period of abstinence. Our dataset is the largest resource of cell types in the mammalian amygdala, with over 163,000 nuclei in our snRNA-seq dataset and 81,000 nuclei in our snATAC-seq dataset (Fig. 2a,b). The snATAC-seq dataset is the first map of cell-type-specific regulatory elements in the amygdala, enabling the identification of TF motifs that may drive addiction-related processes.

Previous rodent snRNA-seq studies have focused on the acute effects of passive treatment with psychoactive drugs^22,23^, which cannot fully capture the motivational processes underlying addiction. In contrast, our behavioral protocol using extended access to cocaine IVSA reflects key aspects of cocaine addiction, including escalation of drug use, enhanced motivation for drug seeking and taking, and persistent drug use despite adverse consequences^76^. In addition, using an outbred rat population allowed us to correlate molecular differences not only with a high AI phenotype, which reflects vulnerability, but also with a low AI phenotype, which reflects resiliency to developing addiction-like behaviors^77^.

One striking finding from our study is that there were strong biases in the direction of regulation of open chromatin regions between high and low AI rats in several main cell types (Supplementary Figs. 16, 17 and 21). Most of these differences were small, suggesting that the combined action of many small effects on gene expression and chromatin accessibility underlies the behavioral differences between rats with high and low AI. Because the HS rats are genetically diverse, the molecular differences between high and low AI rats could arise from genetic differences or it could be a consequence of consuming different amounts of cocaine. The results are consistent with a polygenic model wherein addiction-like behaviors result from the collective action of a large number of genetic risk loci with small individual effects. This is a plausible explanation because of the high genetic diversity in the HS rats and because complex traits, including addiction, are known to be highly polygenic in humans^73,78^. In support of the genetic hypothesis, we observed that most DEGs have eQTLs that were identified independently in HS rat brains^45^ (Supplementary Fig. 17), including *Kcnq3*, *Fkbp5* and *Sgk1* (Fig. 3a–e). Alternatively, a relatively small number of TFs could affect many downstream genes and chromatin sites. Because the motifs with the strongest chromatin accessibility differences (Fig. 5e–h) are recognized by pioneer TFs (for example, BHLH, SOX and FOX) with an intrinsic ability to modify chromatin, they may lead to widespread differences in accessibility^79^. These explanations are not mutually exclusive, and it is probable that some differences are caused by eQTLs while others are caused by differences in the activity of upstream regulators (which themselves may be affected by genetics or other factors).

To uncouple pre-existing genetically controlled gene expression differences from cocaine-induced neuroadaptations, we compared our observed DEGs with differences in expression obtained from genotype-based prediction models. We found significant correlations in observed versus predicted differential gene expression between high versus low AI rats, supporting a genetic role in the differences in gene expression that we observed. The correlation metrics obtained from our analysis were modest, as expected due to three limitations of the predictive model. First, the models are trained on whole-brain tissue lacking the cell-type-specific resolution of our snRNA-seq data. Second, the size of the cohort on which the predictive models were trained was modest (*n* = 339). Third, the models can capture only a small fraction of variation in expression and do not account for trans-acting eQTLs or numerous other influences on gene expression. Despite these limitations, this analysis establishes that at least some of the differences are due to genetic variation (Supplementary Fig. 18). As more rat behavioral GWAS are completed, it will be possible to uncouple the role of genetics versus cocaine exposure more fully, for example, through the use of polygenic risk scores for addiction-related traits^28,30–32,80^.

Consistent with previous findings showing enhanced GABAergic transmission following excessive cocaine use^81^, our differential gene expression analysis showed enrichment of genes in the GABAergic synapse pathway (Fig. 3f) and our electrophysiology results indicated an enhanced GABAergic transmission in high AI rats (Fig. 4b). Moreover, we found that inhibition of GLO1—the enzyme responsible for MG metabolism—restored electrophysiological (Fig. 4c–f) and behavioral (Fig. 4h) differences associated with addiction-like behaviors. Specifically, while pBBG diminished GABA transmission in electrophysiological recordings for both low and high AI rats (Fig. 4c), it had an inhibitory effect on the drug-seeking behaviors in high AI rats but not in low AI rats (Fig. 4h). This suggests that the inhibitory effects of pBBG on relapse-like behaviors depend on a given threshold of GABAergic transmission. These results corroborate previous findings that MG acts as an endogenous competitive agonist for GABA_A_ receptors^82,83^. GABA_A_ receptor agonists used in the context of cocaine-seeking behavior have shown contrasting results leading to both reductions and increases in cocaine-seeking behaviors^84–90^. Since MG is generated in proportion to glycolytic activity of nearly every cell and does not accumulate in synaptic vesicles, it may activate GABA_A_ receptors at synaptic and extra synaptic sites; thus, manipulating the endogenous levels of MG by GLO1 inhibition represents a unique mechanism of GABA_A_ receptor regulation. In our electrophysiological experiments, we did not observe changes in postsynaptic currents in the CeA; thus, we speculate that MG-based pharmacological manipulations may alter presynaptic GABA_A_ receptor function, reducing GABA release at inhibitory terminals and suppressing inhibitory connections in the CeA. Consistent with this notion, previous studies have demonstrated that the activation of presynaptic GABA_B_ receptors suppresses inhibitory connection in the CeA^91^ and that negative regulation of GABAergic transmission can occur through a presynaptic mechanism^92^. An alternative scenario is that the magnitude of effects is not sufficient to cause detectable changes in amplitude. Overall, these results offer a new pharmacological target for improving therapeutic approaches for cocaine addiction.

While the pharmacological inhibition experiments are not cell-type-specific, the pathway enrichment analysis of the transcriptomic data suggest that GABAergic synapse-related genes may be specific to *Cck*^+^/*Vip*^+^ and *Nos*1^+^ subtypes of inhibitory neurons. Previous studies manipulating GLO1 activity directly in the mouse amygdala by transgenic expression of *Glo1* or MG microinjection were sufficient to reduce anxiety-like behaviors^93^. Future experiments targeting specific subregions or cell types of the amygdala will be necessary to further characterize the effects of GLO1 inhibition on cocaine addiction-related phenotypes.

The results from the GLO1 inhibition experiments indicate that an altered metabolic state in the amygdala impacts several cellular processes that are involved in vulnerability to, and development of, addiction. Moreover, genes differentially regulated in high versus low AI rats were enriched in pathways related to energy metabolism, such as oxidative phosphorylation, which determines cellular ATP levels^94^. ATP is not only crucial for sustaining electrophysiological activity and cell signaling in the brain^95,96^, it is also required for ATP-dependent chromatin remodeling events initiated by pioneer TFs^97^. This could potentially explain the striking observations that excitatory and inhibitory neurons show opposite directions of regulation in chromatin accessibility (Supplementary Fig. 21) and that DEGs are enriched in the oxidative phosphorylation pathway (Fig. 3f). Future experiments that directly manipulate the expression of specific metabolic enzymes or pioneer TFs in a cell-type-specific manner will be necessary to fully elucidate their role in addiction.

In conclusion, the amygdalar cellular atlas produced by this study is a valuable resource for understanding the role of cell-type-specific gene regulatory programs in the development of cocaine addiction-related behaviors. Our results emphasize the importance of cellular energetics and GABA_A_-mediated signaling in the enduring effects of cocaine use, and identify GLO1 as a potential new target for the treatment of cocaine addiction.

## Methods

### Experimental

#### Animals

All protocols were approved by the Institutional Animal Care and Use Committee at the University of California San Diego (UCSD). HS rats (RRID:RGD_2314009) were provided by L. Solberg Woods (Wake Forest University School of Medicine). To minimize inbreeding and control genetic drift, the HS rat colony is maintained using an optimized breeding strategy, with each breeder pair contributing one male and one female to subsequent generations. Rats were shipped at 3–4 weeks of age, quarantined for 2 weeks and then housed two per cage on a 12 h/12 h reversed light/dark cycle in a temperature- (20–22 °C) and humidity- (45–55%) controlled vivarium with ad libitum access to tap water and food pellets. Rats were 3–4 weeks of age at the start of the experiment. We used 46 HS rats for the behavioral experiments presented in Fig. 1, of which 20 male rats (high and low AI) were used to generate snRNA-seq and snATAC-seq data, along with 11 naive male rats. Additionally, 26 of these 46 behaviorally phenotyped rats (13 female, 13 male) were used for the cue-induced reinstatement experiments. For snRNA-seq, we used 19 male rats (6 high AI, 6 low AI, 7 naive). For snATAC-seq, we used 12 male rats (4 high AI, 4 low AI, 4 naive). We used a different cohort of 15 female and male HS rats (5 high AI, 5 low AI, 5 naive) for the electrophysiology experiments. In addition, 6 male ACI/EurMcw rats (RRID:RRRC_00284) were obtained from the Rat Genome Database and used for snRNA/snATAC-seq. No statistical methods were used to predetermine sample sizes, but our sample sizes are similar to those reported in previous publications^99,100^.

#### Drugs

Cocaine HCl (National Institute on Drug Abuse) was dissolved in 0.9% saline and administered intravenously at a dose of 0.5 mg kg^–1^ per infusion. pBBG was synthesized in the laboratory of D. Siegel (Skaggs School of Pharmacy and Pharmaceutical Sciences, UCSD). pBBG was dissolved in a vehicle of 8% dimethylsulfoxide, 18% Tween-80 and 74% distilled water and administered intraperitoneally 30 min before the test session.

#### Brain samples

Brain tissues were obtained by the cocaine brain bank at UCSD^24^ after 4 weeks of abstinence from cocaine self-administration (SA), a timepoint used in previous studies to examine long-lasting effects of SA^32,101–106^. Behavioral data was collected with the MedPCIv v.5 software. Brain tissues were extracted and snap-frozen (at –30 °C). Cryosections of 500 μm (Bregma –1.80 to 3.30 mm) were used to dissect the amygdala, including the CeA, BLA and medial amygdala from both hemispheres. Punches from three sections were combined for each rat. In addition, six ACI/EurMcw rats were used for dissection of the CeA and BLA.

#### Single-cell library preparation, sequencing and alignment

snRNA-seq libraries from whole amygdala tissues were generated by the Center for Epigenomics, UCSD. Briefly, frozen tissue was homogenized via glass dounce. Nuclei were resuspended in 500 µl of nuclei permeabilization buffer (0.1% Triton-X-100 (Sigma-Aldrich, catalog no. T8787), 1× protease inhibitor, 1 mM DTT and 1 U µl^–1^ RNase inhibitor (Promega, catalog no. N211B), 2% bovine serum albumin (BSA; Sigma-Aldrich, catalog no. SRE0036) in PBS). Samples were incubated on a rotator for 5 min at 4 °C and then centrifuged at 500*g* for 5 min (4 °C). Pellets were resuspended in 400 µl of sort buffer (1 mM EDTA, 0.2 U µl^–1^ RNase inhibitor (Promega, catalog no. N211B), 2% BSA (Sigma-Aldrich, catalog no. SRE0036) in PBS) and stained with DRAQ7 (1:100; Cell Signaling, catalog no. 7406). Up to 75,000 nuclei were sorted using a SH800 sorter (Sony Cell Sorter Software v.2.1.2-5) into 50 µl of collection buffer consisting of 1 U µl^–1^ RNase inhibitor in 5% BSA. Sorted nuclei were centrifuged at 1,000*g* for 15 min at 4 °C and then resuspended in 35 µl of reaction buffer (0.2 U µl^–1^ RNase inhibitor (Promega, catalog no. N211B) and 2% BSA (Sigma-Aldrich, catalog no. SRE0036) in PBS). Then, 12,000 nuclei were loaded onto a Chromium Controller (10x Genomics). Libraries were generated using the Chromium Single-Cell 3′ Library Construction Kit v.3 (10x Genomics, catalog no. 1000075) with the Chromium Single-Cell B Chip Kit (10x Genomics, catalog no. 1000153) and the Chromium i7 Multiplex Kit for sample indexing (10x Genomics, catalog no. 120262) according to manufacturer specifications. cDNA was amplified for 12 PCR cycles.

For snATAC-seq libraries from the whole amygdala tissues, nuclei were purified using an established method^107^. Frozen amygdala tissue was homogenized using a 2 ml glass dounce with 1 ml cold homogenization buffer (0.26 M sucrose, 0.03 M KCl, 0.01 M MgCl_2_, 0.02 M Tricine-KOH pH 7.8, 0.001 M DTT, 0.5 mM spermidine, 0.15 mM spermine and 0.3% NP40). The cell suspension was filtered using a 70 μm Flowmi strainer (Millipore Sigma, catalog no. BAH136800070) and centrifuged at 350*g* for 5 min at 4 °C. Nuclei were isolated by iodixanol (Millipore Sigma, catalog no. D1556) density gradient. The nuclei iodixanol solution (25%) was layered on top of 40% and 30% iodixanol solutions. Samples were centrifuged in a swinging bucket centrifuge at 3,000*g* for 20 min at 4 °C. Nuclei were isolated from the 30–40% interface. Nuclei were washed in ATAC-RSB-Tween buffer (0.01 M Tris-HCl pH 7.5, 0.01 M NaCl, 0.003 M MgCl_2_, 0.1% Tween-20) and then resuspended in nuclei resuspension buffer (10x Genomics, catalog no. PN 2000207). Then, 12,000 nuclei were loaded on the 10x Genomics Chromium Controller for GEM (gel bead in emulsion) generation. Libraries were generated using the Chromium Next GEM Single Cell ATAC v.1.1 (10x Genomics, catalog no. PN-1000175) with the Chromium Next GEM Chip H Single Cell Kit (10x Genomics, catalog no. 1000162) and the Chromium i7 Multiplex Kit for sample indexing (10x Genomics, catalog no. 1000212) according to manufacturer specifications. DNA was amplified for eight cycles.

For snRNA libraries from BLA and CeA, frozen brain tissues were obtained from the ACI/EurMcw rat strain—one of the HS rat founder strains. Nuclei were isolated as described above for snATAC-seq libraries. RNAse inhibitors (Roche Diagnostics, catalog no. 03335402001) were added to all buffers (1 U μl^–1^). Then, 12,000 nuclei were loaded on the 10x Genomics Chromium Controller for GEM generation. Libraries were generated using the Chromium Next GEM Single Cell Multiome Reagent Kit A (catalog no. 1000282) following Chromium Next GEM Single Cell Multiome ATAC + Gene Expression Reagent Kits User Guide (10x Genomics). After the transposition reaction, nuclei were encapsulated and barcoded. Next-generation sequencing libraries were constructed following the User Guide. Final libraries were sequenced using the NovaSeq6000 (Illumina).

#### Behavioral experiments

Behavioral testing of rats used for snRNA-seq and snATAC-seq experiments followed an established protocol^24,33,34^. Briefly, after surgical implantation of intravenous catheters and a week of recovery, HS rats were trained to self-administer cocaine (fixed ratio 1 with 0.5 mg kg^–1^ per infusion) in ten ShA sessions (2 h per day, 5 days per week). Next, the animals were allowed to self-administer cocaine in 14 LgA sessions (6 h per day, 5 days per week) to measure the escalation of drug intake (Fig. 1e). Then, rats were screened for motivation using the PR test and for persistent drug-seeking despite adverse consequences using footshock (30% contingency). The breakpoint (Fig. 1f) was defined as the maximal number of presses completed before a 60-min period during which a rat does not complete the next schedule. Rats were classified as high AI or low AI via a median split^108,109^. AI was computed by averaging normalized measurements (*z*-scores) for the three behavioral tests after the LgA phase: escalation of drug intake, motivation and compulsive-like behavior, or drug taking despite adverse consequences (Fig. 1c,d)^33^. The *z*-scores were calculated as *z* = (*x* – *μ*)/*σ*, where *x* is the raw value, *μ* is the mean of the cohort and *σ* is the s.d. of the cohort. For the pBBG studies (Fig. 4h), we used a different cohort of rats with low or high AI phenotyped using the same behavioral protocol. Four weeks after the last IVSA session, the rats were placed back in the SA chambers without the availability of cocaine. The number of responses to the previous drug-paired lever (cocaine-seeking behavior) was measured 30 min after intraperitoneal injection of pBBG (15 mg kg^–1^ ml^–1^) or its vehicle, in a Latin square design. The 30-min timepoint was selected based on a previous study^57^. Data were analyzed using Prism v.9.0 software (GraphPad). SA data were analyzed using repeated-measures ANOVA or mixed effect model followed by Bonferroni post hoc tests when appropriate. For pairwise comparisons, data were analyzed using the unpaired *t*-test. Data are expressed as mean ± s.e.m. unless otherwise specified. Values of *P* < 0.05 were considered statistically significant. Data distributions were assumed to be normal, but this was not tested formally. Experimenters were blinded to group allocation during behavioral data collection before brain collection.

#### Electrophysiology

CeA slices were prepared after 4 weeks of abstinence from cocaine IVSA following the same behavioral protocol described above or age-matched naive rats that received sham IV surgery. These rats were distinct from those used for snRNA-seq and snATAC-seq and included five high AI, five low AI and five naive rats. Slices from each group were also used to record sIPSCs after pBBG treatment. Brain tissues were placed in oxygenated (95% O_2_, 5% CO_2_) ice-cold cutting solution (206 mM sucrose, 2.5 mM KCl, 1.2 mM NaH_2_PO_4_, 7 mM MgCl_2_, 0.5 mM CaCl_2_, 26 mM NaHCO_3_, 5 mM glucose and 5 mM Hepes). Transverse slices (300 μm thick) were cut on a Vibratome (Leica VT1200S; Leica Microsystems) and transferred to oxygenated artificial cerebrospinal fluid (130 mM NaCl, 2.5 mM KCl, 1.2 mM NaH_2_PO_4_, 2.0 mM MgSO_4_·7H_2_O, 2.0 mM CaCl_2_, 26 mM NaHCO_3_ and 10 mM glucose) for 30 min at 35 °C and then at room temperature for the rest of the experiment conducted in a recording chamber mounted on the stage of an upright microscope (Olympus, catalog no. BX50WI). The slices were perfused continuously with oxygenated artificial cerebrospinal fluid at a rate of 3 ml min^–1^. Whole-cell recordings were performed using a Multiclamp 700B amplifier (10 kHz sampling rate, 10 kHz low-pass filter) and Digidata 1440A and pClamp 10 software (Molecular Devices). Patch pipettes (4–6 MΩ) were pulled from borosilicate glass (Warner Instruments) and filled with 70 mM KMeSO_4_, 55 mM KCl, 10 mM NaCl, 2 mM MgCl_2_, 10 mM Hepes, 2 mM Na-ATP and 0.2 mM Na-GTP. Pharmacologically isolated sIPSCs were recorded in the presence of the glutamate receptor blockers, CNQX (Tocris, catalog no. 0190) and APV (Tocris, catalog no. 189) and the GABA-B receptor antagonist CGP55845 (Tocris, catalog no. 1246). Experiments with a series resistance of >15 MΩ or >20% change in series resistance were excluded from the final dataset. pBBG (2.5 μM) was acutely applied in the bath. The frequency, amplitude and kinetics of sIPSCs were analyzed using semiautomated threshold-based minidetection software (Easy Electrophysiology) and confirmed visually. Data were analyzed using Prism v.9.0 software (GraphPad) with one-way ANOVA followed by post hoc Tukey honestly significant difference test or with paired *t*-tests. Data are expressed as mean ± s.e.m unless otherwise specified. Values of *P* < 0.05 were considered statistically significant.

### Computational

#### Alignment of snRNA-seq and snATAC-seq reads

FASTQ files were generated from binary base call files using Cell Ranger v.3.1.0 for snRNA-seq data, Cell Ranger ATAC v.2.0.0 for snATAC-seq data and Cell Ranger ARC v.2.0.0 for Multiome data, using the mkfastq command^110,111^. Reads were aligned to a custom rn6 reference genome created from FASTA and genome annotation files for *Rattus norvegicus* Rnor_6.0 (Ensembl release 98)^112^ and JASPAR2022 motifs^113^, using Cell Ranger’s count command.

#### Quality control and preprocessing of snRNA-seq data

All snRNA-seq preprocessing was performed with Seurat v.3.2.3 (ref. ^114^). For each sample, we computed metrics for each cell including the number of unique genes detected (nFeature_RNA); the total molecules detected (nCount_RNA) and the percentage of reads mapping to the mitochondrial genome (percent.mt) (Supplementary Figs. 1–3, Supplementary Data 1 and Supplementary Data 3). We removed cells for which any of these metrics was more than three s.d. units from the mean in the sample. Next, we normalized the count data for each sample using sctransform^115^ with percent.mt as a covariate.

#### Integrating snRNA-seq data across samples and clustering

To integrate snRNA-seq data across samples, we used reciprocal principal component analysis (PCA), as implemented in Seurat^114,116^. We identified 2,000 highly variable features across the samples using the ‘SelectIntegrationFeatures()’ function, performed dimensionality reduction with PCA on each sample, and identified anchors using ‘FindIntegrationAnchors()‘, specifying reciprocal PCA as the dimensionality reduction method. We used the resulting anchor set to integrate across samples using ‘IntegrateData()’ with two rats (one high AI, one low AI) as reference samples. We clustered the integrated dataset by constructing a K-nearest neighbor (KNN) graph using the first 30 principal components followed by the Louvain algorithm. Finally, we ran PCA on the integrated dataset and visualized the data in two dimensions using uniform manifold approximation and projection (UMAP) (Supplementary Fig. 9a–c). To compare CeA and BLA subregion samples with the whole amygdala, we subsampled whole amygdala samples from the naive rats and performed the same integration technique. The integrated subregion data was visualized using UMAP.

#### Cell-type assignment for snRNA-seq data

We identified marker genes of each cluster in our integrated snRNA-seq dataset using MAST^117^, implemented with the ‘FindMarkers()’ function in Seurat. Cell type identities were assigned based on expression of known marker genes.

#### Cell-type-specific gene expression analysis for snRNA-seq data

We tested for cell-type-specific DEGs between high versus low AI rats using the negative binomial test^59,60^ implemented with the ‘FindMarkers()’ function in Seurat, using percent.mt and the library prep date as covariates. We did not pre-filter genes for testing based on logFC or minimum fraction of cells in which a gene was detected. We used Benjamini–Hochberg FDR of 10% as a significance threshold. Permutation tests were performed using the same methods, covariates and filtering options but with shuffled AI labels. Results from permuted and unpermuted data were compared by visualizing the distributions of uncorrected *P* values (Supplementary Fig. 15 and Supplementary Data 6).

We used ClusterProfiler^118^ to perform GSEA of KEGG pathways. A ranked list of the avg_logFC values for all genes evaluated with our negative binomial test was given as input to GSEA. Multiple testing correction for GSEA results was performed using Benjamini–Hochberg adjustment, with statistical significance assessed at FDR < 10%.

Conditionally independent cis-eQTLs (FDR < 5%) were downloaded from the RatGTEx portal (https://ratgtex.org/download/). We examined cis-eQTLs in the following brain tissues: BLA, brain, infralimbic, lateral habenula, nucleus accumbens, nucleus accumbens 2, orbitofrontal cortex, prelimbic, prelimbic 2. We assessed enrichment of significant DEGs (FDR < 10%) that also had eQTLs in the rat brain with a Chi-squared test.

To obtain bootstrap distributions of DEG effect sizes, we resampled nuclei with replacement 1,000 times. Resampling was performed separately for nuclei from high and low AI rats so that the sample size of each set remained consistent. For each bootstrap iteration, we recorded the *P* value and the coefficients for the high and low AI conditions from the negative binomial regression performed by Seurat’s ‘FindMarkers()’ function. We then rescaled the coefficient to be in units of log2FC. The log2FC estimates obtained by this method differ slightly from Seurat’s average (avg)_log2FC estimates, which introduce a pseudocount and do not use covariates. The distribution of resulting bootstrap FC estimates and *Q* values were visualized with violin plots (Fig. 3c–e).

#### Comparing observed gene expression differences with predicted gene expression differences based on cis-genetic variation

To estimate the genetic component of gene expression variation in the brain, conditionally independent cis-eQTLs and their allelic FC estimates for whole-brain hemisphere tissue were downloaded from the RatGTEx Portal (https://ratgtex.org/download/). Using allelic FC as effect size, gene expression was predicted from genotypes using eQTL linear models^51^ (https://github.com/PejLab/gene_expr_pred). Predicted relative expression was obtained for 26 rats with genotypes, for genes with at least one significant cis-eQTL. Genes with zero-variance predictions were removed, resulting in predictions for 8,997 genes. To estimate prediction accuracy, Pearson correlation *r*^2^ was calculated between predicted and observed log-TPM expression for the 339 rats used to discover whole-brain-hemisphere eQTLs. We compared differences in mean predicted expression between high and low AI rats to observed avg_logFC estimates from our DEG analysis by computing Spearman correlation (*ρ*) at different prediction accuracy (*r*^2^) cutoffs (Supplementary Table 3). Spearman correlation confidence intervals (CIs) were calculated using $\tan h \left(\tan {h}^{-1}(\,\rho )\pm \frac{1.96}{\sqrt{N-3}}\right)$.

#### Quality control and preprocessing of snATAC-seq data

All snATAC-seq data preprocessing was performed with MACS2 (ref. ^58^) and Signac^119^. We called peaks separately using MACS2 because Cell Ranger’s peak calling function can merge distinct peaks into single regions^119^. We first called peaks on the snATAC-seq BAM files for each rat with MACS2 (‘macs2 callpeak -t {input} -f BAM -n {sample} --outdir {output} {params} --nomodel --shift -100 --ext 200 --qval 5e-2 -B --SPMR’). We confirmed that MACS2 calls more peaks and peaks with smaller widths compared with Cell Ranger (Supplementary Fig. 25) and merged overlapping peaks to generate a combined peak set using BEDtools^120^ (‘bedtools merge’). Using Signac, we generated a new peak by barcode matrix for each sample using the ‘FeatureMatrix()’ function, created ChromatinAssay objects in Signac with the BSgenome.Rnorvegicus.UCSC.rn6 (ref. ^121^) reference genome using the ‘CreateChromatinAssay()’ function and created Seurat objects with the ‘CreateSeuratObject()’ function. We computed quality control metrics for each sample, including nucleosome banding pattern (nucleosome_signal), TSS enrichment score (TSS.enrichment), total fragments in peaks (peak_region_fragments) and fraction of fragments in peaks (pct_reads_in_peaks) (Supplementary Fig. 4–6, Supplementary Data 2 and Supplementary Data 4). We removed cells where any of these metrics was more than two s.d. units from the mean in the sample. We removed one rat (FTL_463_M757_933000320046135) from our dataset due to the very low number of detected fragments per cell in this sample (Supplementary Fig. 26).

#### Integrating snATAC-seq data across samples and clustering

Each sample was normalized using term frequency-inverse document frequency followed by singular value decomposition^119^. The combined steps of term frequency-inverse document frequency followed by singular value decomposition are known as latent semantic indexing^122,123^. Nonlinear dimensionality reduction and clustering were performed using UMAP and KNN, respectively, following the same procedure as for the snRNA-seq data. We merged the data across all samples, repeated the process of latent semantic indexing and integrated the merged dataset using Harmony^124^. We observed successful reduction of batch effects following integration (Supplementary Fig. 9d–f). We then performed nonlinear dimensionality reduction and clustering with UMAP and KNN. Raw counts were used for downstream differential accessibility analyses.

#### Label transfer and cell-type assignment for snATAC-seq data

We created a gene activity matrix for the integrated snATAC-seq data using the ‘GeneActivity()’ function in Signac. This uses the number of fragments per cell overlapping the promoter region of a given gene to calculate a gene activity score. Gene activity scores were normalized using the ‘NormalizeData()’ function in Seurat with the normalization method set to ‘LogNormalize’ and the scaling factor set to the median value of nCount_RNA across all cells, calculated from the gene activity scores. Cell type identities were assigned by integrating the snATAC-seq data with the integrated snRNA-seq data and performing label transfer^114^. This process returns a classification score for each cell for each cell type defined in the scRNA-seq data. Each cell was assigned the cell-type identity with the highest score. By identifying matched cells in the snRNA-seq dataset, we were able to impute RNA expression values for each cell in our snATAC-seq dataset.

#### Differential chromatin accessibility analysis of snATAC-seq data

To identify DA genomic regions between high versus low AI rats, we applied the negative binomial test^115,125^ implemented in Seurat’s ‘FindMarkers()’ function using raw snATAC-seq counts as input and peak_region_fragments, library batch date and rat sample identification number as covariates. Multiple testing correction was performed using the Benjamini–Hochberg method and FDR < 10% was used to determine statistical significance. Permutation tests were performed in the same manner as the differential gene expression analyses.

#### Partitioned heritability analysis

We downloaded summary statistics for the 2019 GWAS of tobacco and alcohol use by Liu et al.^74^ and used the munge_sumstats.py script from LD score (LDSC)^73^ to reformat the file. We used the significant differential peaks (FDR < 10%) for each cell type as foreground peaks and DNase I hypersensitivity profiles for 53 epigenomes from ENCODE Honeybadger2 as background peaks. We used the University of California Santa Cruz (UCSC) liftOver tool to convert the foreground peaks from rn6 to hg19. We used the make_annot.py script to make annotation files, the ldsc.py script to compute annotation-specific LD scores and the European 1000 Genomes Phase 3 PLINK^126^ files to compute LD scores. Finally, using the baseline model and standard regression weights from the LDSC Partitioned Heritability tutorial, we ran a cell-type-specific partitioned heritability analysis.

#### Fisher’s exact tests

We performed FETs to test (1) whether differential peaks of chromatin accessibility (FDR < 10%) were enriched in promoter regions compared with other genomic regions and (2) whether significant DEGs (FDR < 10%) were enriched for promoters with significant differential chromatin accessibility. For the first FET, we used the annotatePeaks.pl script from HOMER to annotate accessible chromatin regions and significant differential peaks (FDR < 10%) for each cell type in our integrated dataset^127^. For each cell type, we generated a 2 × 2 contingency table for the FET where the cells contained the following counts: differential peaks with a TSS/promoter annotation; differential peaks without a TSS/promoter annotation; nondifferential peaks (FDR > 10%) with a TSS/promoter annotation and nondifferential peaks (FDR > 10%) without a TSS/promoter annotation. For the second FET, we obtained gene coordinates from the TxDb.Rnorvegicus.UCSC.rn6.refGene annotation package and defined promoter regions as –1,500 bases upstream to +500 bases downstream of the TSS. We then generated a 2 × 2 contingency table for the FET that contained the number of DEGs with DA promoters, DEGs with non-DA promoters, non-DEGs with DA promoters and non-DEGs with non-DA promoters.

#### Measuring differential activity of TFs with chromVAR

We measured cell-type-specific motif activities using chromVAR to test for per motif deviations in accessibility between nuclei from high versus low AI rats. Motif data was pulled from the JASPAR2020 database and integrated with snATAC-seq data using the ‘AddMotifs()’ function in Signac. After adding motifs to our snATAC-seq dataset, we ran the ‘RunChromVAR()’ wrapper in Signac. Differential analysis of chromVAR deviation scores was performed using the Wilcoxon rank-sum test between high versus low AI rats in each cell type. Differential testing was performed using Seurat’s ‘FindMarkers()’ function with the mean function set as ‘rowMeans()’ to calculate average difference in deviation scores between groups. Multiple testing correction was performed using Benjamini–Hochberg adjustment and FDR < 10% was used to determine statistical significance. Motif clusters were defined using the cluster numbers from JASPAR’s matrix clustering-results and the names of the clusters were annotated manually. When selecting clusters to visualize, we retrieved the top 50 most significant motifs (FDR < 10%) per cell type, highlighting the motif clusters present.

### Reporting summary

Further information on research design is available in the Nature Portfolio Reporting Summary linked to this article.

## Online content

Any methods, additional references, Nature Portfolio reporting summaries, source data, extended data, supplementary information, acknowledgements, peer review information; details of author contributions and competing interests; and statements of data and code availability are available at 10.1038/s41593-023-01452-y.

### Supplementary information

Supplementary Information Supplementary Figs. 1–26 and Tables 1–6.

Reporting Summary

Supplementary Data 1 List of snRNA-seq rat samples included in analysis, their AIs, batch information and Cell Ranger summary metrics.

Supplementary Data 2 List of snATAC-seq rat samples included in analysis, their AIs, batch information and Cell Ranger summary metrics.

Supplementary Data 3 Per nucleus metrics for all nuclei in snRNA-seq dataset after filtering. This table contains selected columns from the metadata table for the Seurat object containing the integrated snRNA-seq data.

Supplementary Data 4 Per nucleus metrics for all nuclei in snATAC-seq dataset after filtering. This table contains selected columns from the metadata table for the Signac object containing the integrated snATAC-seq data.

Supplementary Data 5 All cell-type-specific differential gene expression analysis results, obtained using the negative binomial test.

Supplementary Data 6 Results of permutation test for differential gene expression analysis using negative binomial test.

Supplementary Data 7 All DEGs (FDR < 10%) that also have eQTLs in the rat brain, with a list of variant IDs for corresponding eQTLs.

Supplementary Data 8 KEGG GSEA results.

Supplementary Data 9 All cell-type-specific differential peak accessibility analysis results, obtained using the negative binomial test.

Supplementary Data 10 Permutation test for differential peak accessibility analysis results using negative binomial test.

Supplementary Data 11 Peak annotations for all OCRs in integrated snATAC-seq dataset.

Supplementary Data 12 ChromVar analysis results.

## Data Availability

The datasets generated in the current study are available through the Gene Expression Omnibus (GSE212417). The following publicly available datasets were used: *Rattus norvegicus* Ensembl v.98 reference genome and genome assembly (Rnor_6.0, http://useast.ensembl.org/Rattus_norvegicus/Info/Index); JASPAR2022 TF binding profiles for vertebrates (https://jaspar.genereg.net/); ENCODE Honeybadger2 ChIP–seq (https://personal.broadinstitute.org/meuleman/reg2map/); Liu et al.^74^ GWAS for tobacco and nicotine addiction summary statistics; RatGTEx Portal tissue-specific cis-eQTLs (https://ratgtex.org/download/); 1000 Genomes European reference panel (https://alkesgroup.broadinstitute.org/LDSCORE/); KEGG pathways (https://www.kegg.jp/kegg/rest/keggapi.html). The HS rats genotype, predicted gene expression and behavioral data are available through the Zenodo repository (10.5281/zenodo.8242458).

## Code availability

All code used to perform analyses in this paper can be found at https://github.com/mcvickerlab/sn_cocaine_rats.
