# Author Correction: Single-nucleus genomics in outbred rats with divergent cocaine addiction-like behaviors reveals changes in amygdala GABAergic inhibition

**DOI:** 10.1038/s41593-023-01489-z

**Published:** 2023-10-16

**Authors:** Jessica L. Zhou, Giordano de Guglielmo, Aaron J. Ho, Marsida Kallupi, Narayan Pokhrel, Hai-Ri Li, Apurva S. Chitre, Daniel Munro, Pejman Mohammadi, Lieselot L. G. Carrette, Olivier George, Abraham A. Palmer, Graham McVicker, Francesca Telese

**Affiliations:** 1 Bioinformatics and Systems Biology Program, University of California San Diego, La Jolla, CA USA; 2 Integrative Biology Laboratory, Salk Institute for Biological Studies, La Jolla, CA USA; 3 Department of Psychiatry, University of California, San Diego, La Jolla, CA USA; 4 Department of Medicine, University of California San Diego, La Jolla, CA USA; 5 Department of Integrative Structural and Computational Biology, The Scripps Research Institute, La Jolla, CA USA; 6 Center for Immunity and Immunotherapies, Seattle Children’s Research Institute, Seattle, WA USA; 7 Department of Pediatrics, University of Washington School of Medicine, Seattle, WA USA; 8 Department of Genome Sciences, University of Washington, Seattle, WA USA; 9 Institute for Genomic Medicine, University of California San Diego, La Jolla, CA USA

**Keywords:** Epigenetics and behaviour, Reward, Addiction, Bioinformatics, RNA sequencing

Correction to: *Nature Neuroscience* 10.1038/s41593-023-01452-y, published online 5 October 2023.

In the version of this article initially published, there was an error in the title: “changes in amygdala GABAergic inhibition” first appeared as “changes in gene amygdala GABAergic inhibition”. The error has been corrected in the HTML and PDF versions of the article.

