# Supplementary material for: Single-nucleus genomics in outbred rats with divergent cocaine addiction-like behaviors reveals changes in amygdala GABAergic inhibition
Source: Nat Neurosci. 2023 Oct 5;26(11):1868–79. doi: 10.1038/s41593-023-01452-y (PMC10620093; doi:10.1038/s41593-023-01452-y)
Supplement: Supplementary file 1 — Supplementary Figs. 1–26 and Tables 1–6. [file 41593_2023_1452_MOESM1_ESM.pdf]

nature neuroscience

Article https://doi.org/10.1038/s41593-023-01452-y

Single-nucleus genomics in outbred rats with divergent cocaine addiction-like behaviors reveals changes in amygdala GABAergic inhibition

In the format provided by the authors and unedited

Supplementary Information

Supplementary Figures

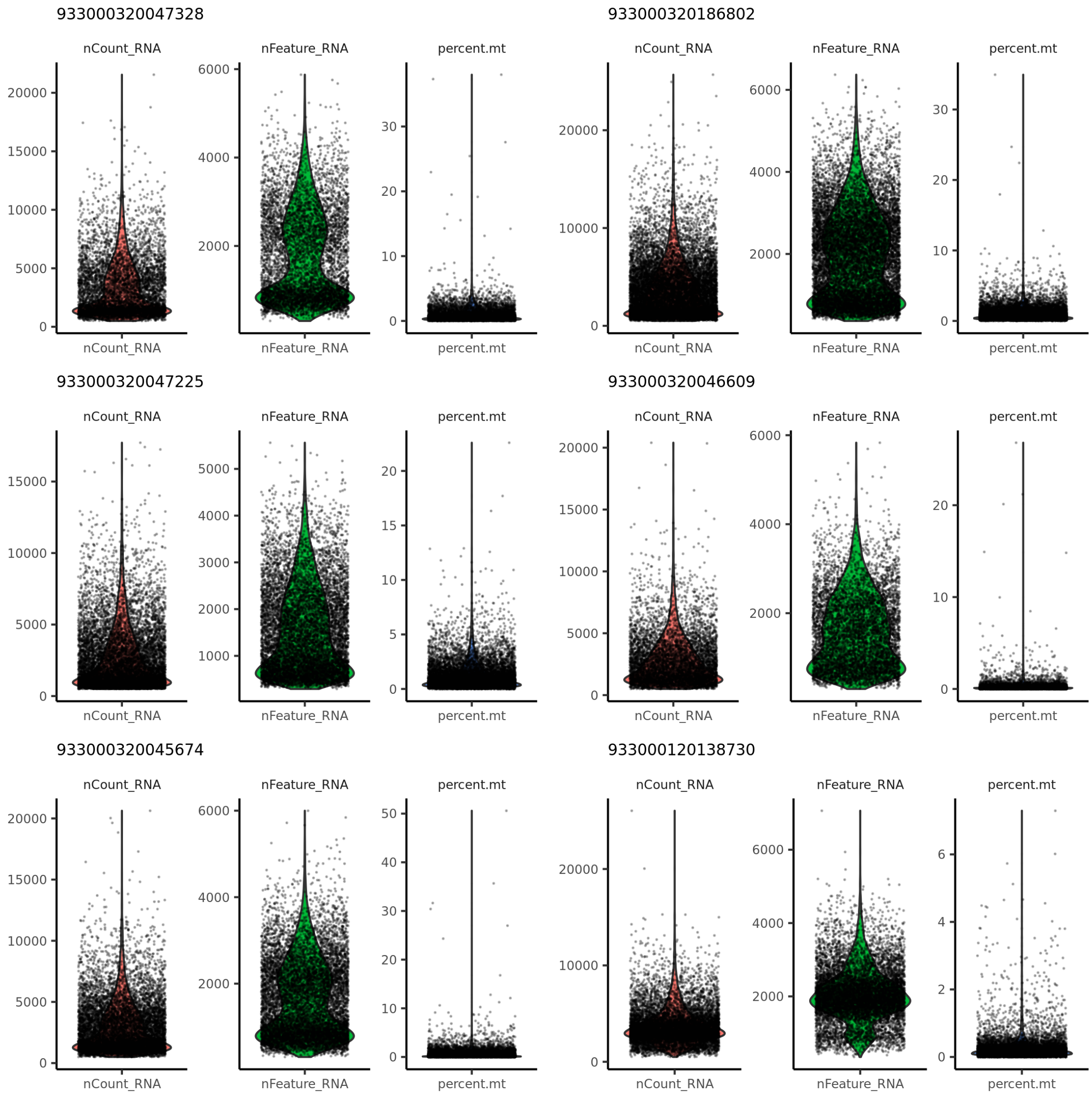

Supplementary Figure 1. snRNA-seq profiles of 6 high AI rats. For each rat the number of unique genes detected per cell (nFeature_RNA), total number of reads within each cell (nCount_RNA), and percentage of percent mitochondrial reads are shown for each cell. Quality metrics calculated with Seurat.

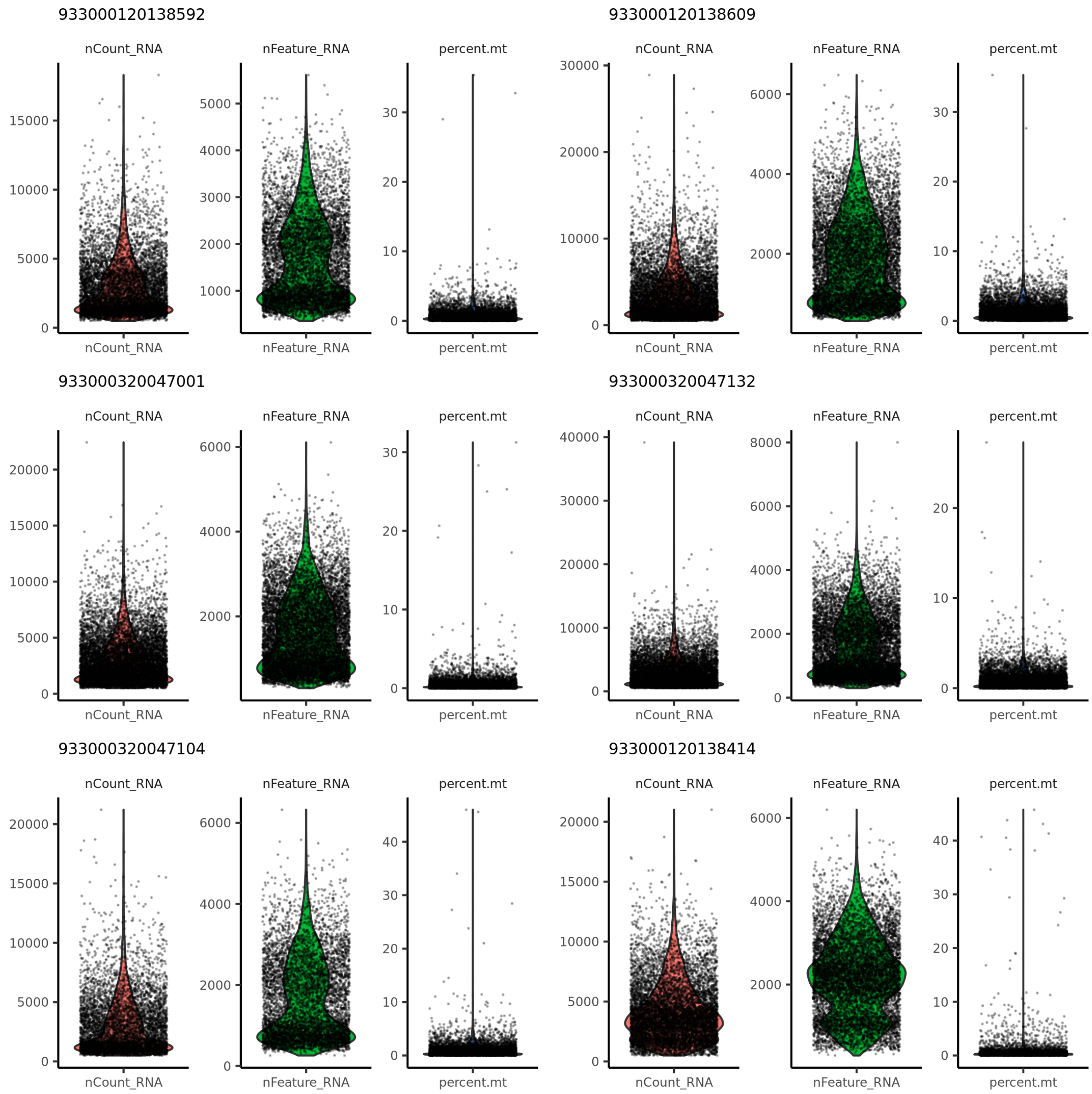

Supplementary Figure 2. snRNA-seq profiles of 6 low AI rats. For each rat the number of unique genes detected per cell (nFeature_RNA), total number of reads within each cell (nCount_RNA), and percentage of percent mitochondrial reads are shown for each cell. Quality metrics calculated with Seurat.

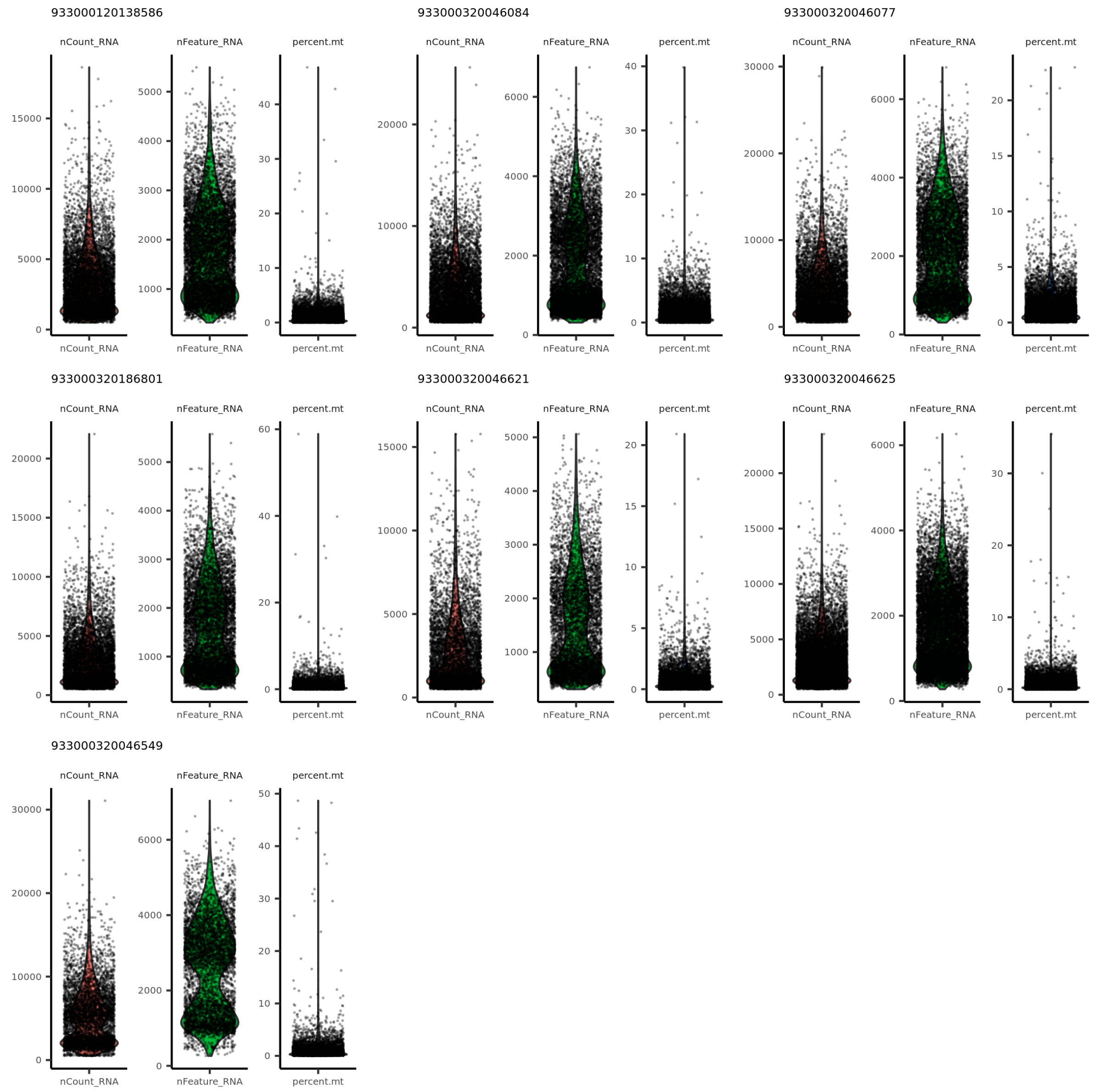

Supplementary Figure 3. snRNA-seq profiles of 7 naive rats. For each rat the number of unique genes detected per cell (nFeature_RNA), total number of reads within each cell (nCount_RNA), and percentage of percent mitochondrial reads are shown for each cell. Quality metrics calculated with Seurat.

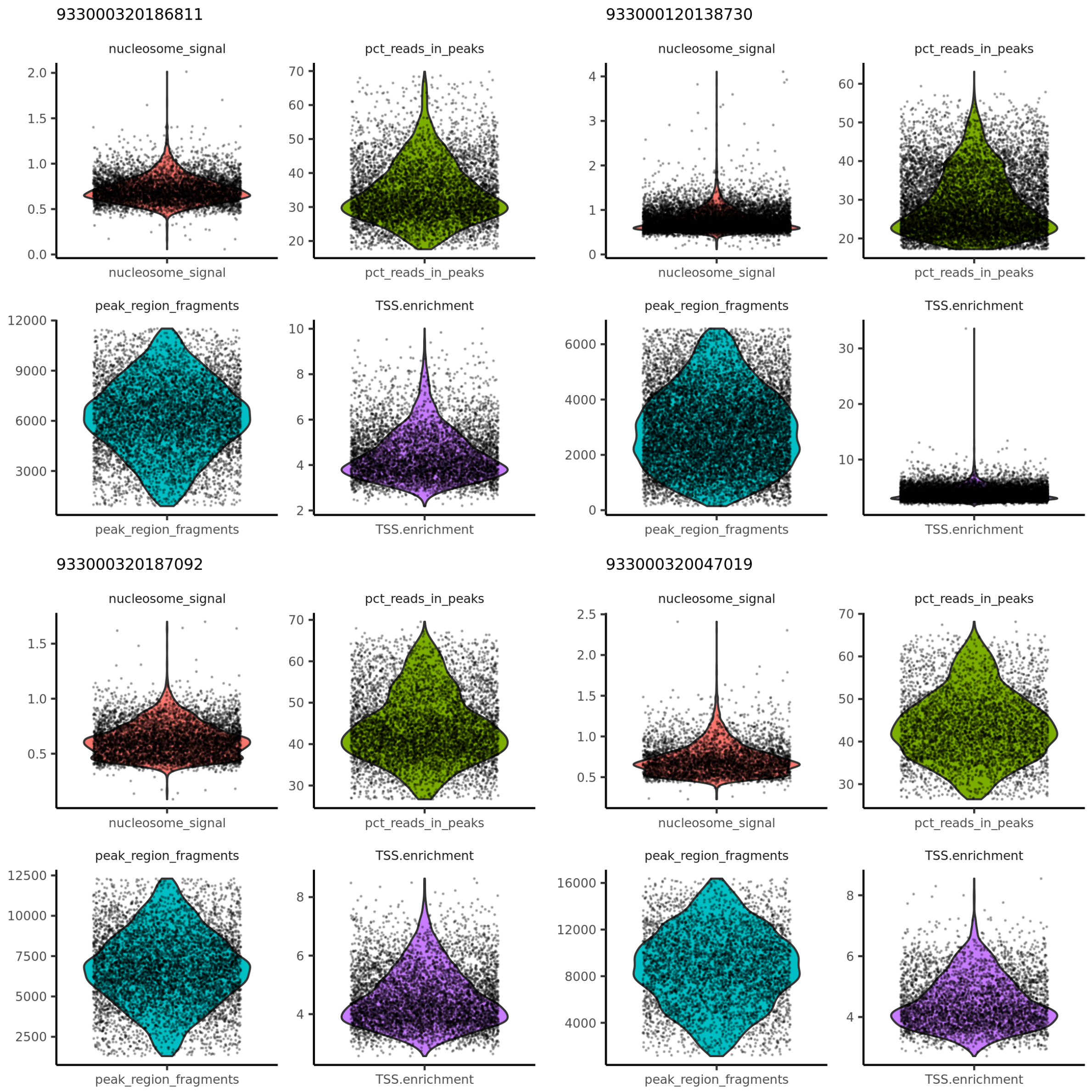

Supplementary Figure 4. snATAC-seq profiles of 4 high AI rats. For each rat the ratio of mononucleosomal to nucleosome-free fragments (nucleosome_signal), percentage of fragments that fall within ATAC-seq peaks (pct_reads_in_peaks), total number of fragments in peaks (peak_region_fragments), and transcription start site enrichment score (TSS.enrichment) are shown for each cell. Quality metrics calculated with Signac.

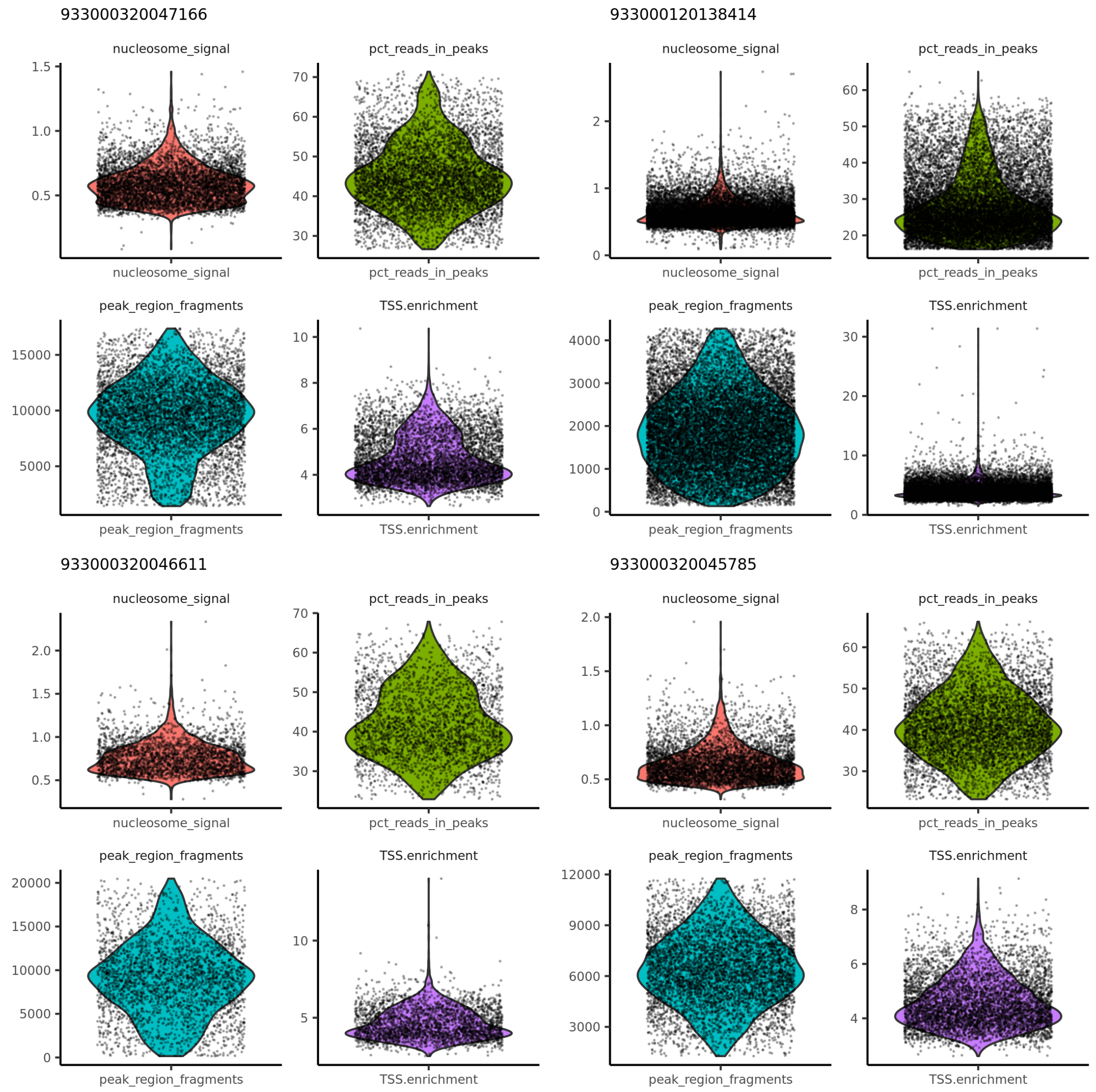

Supplementary Figure 5. snATAC-seq profiles of 4 low AI rats. For each rat the ratio of mononucleosomal to nucleosome-free fragments (nucleosome_signal), percentage of fragments that fall within ATAC-seq peaks (pct_reads_in_peaks), total number of fragments in peaks (peak_region_fragments), and transcription start site enrichment score (TSS.enrichment) are shown for each cell. Quality metrics calculated with Signac.

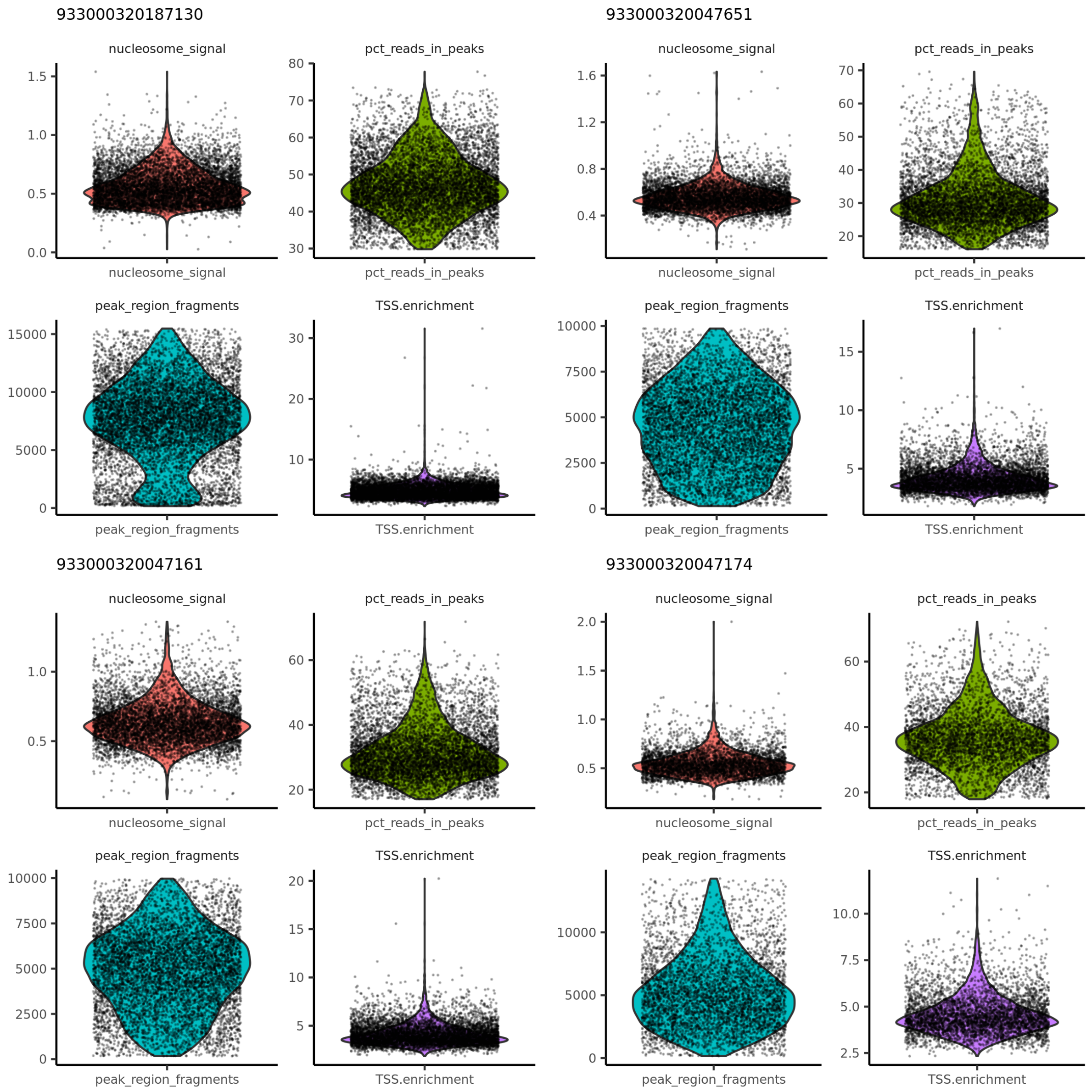

Supplementary Figure 6. snATAC-seq profiles of 4 low naive rats. For each rat the ratio of mononucleosomal to nucleosome-free fragments (nucleosome_signal), percentage of fragments that fall within ATAC-seq peaks (pct_reads_in_peaks), total number of fragments in peaks (peak_region_fragments), and transcription start site enrichment score (TSS.enrichment) are shown for each cell. Quality metrics calculated with Signac.

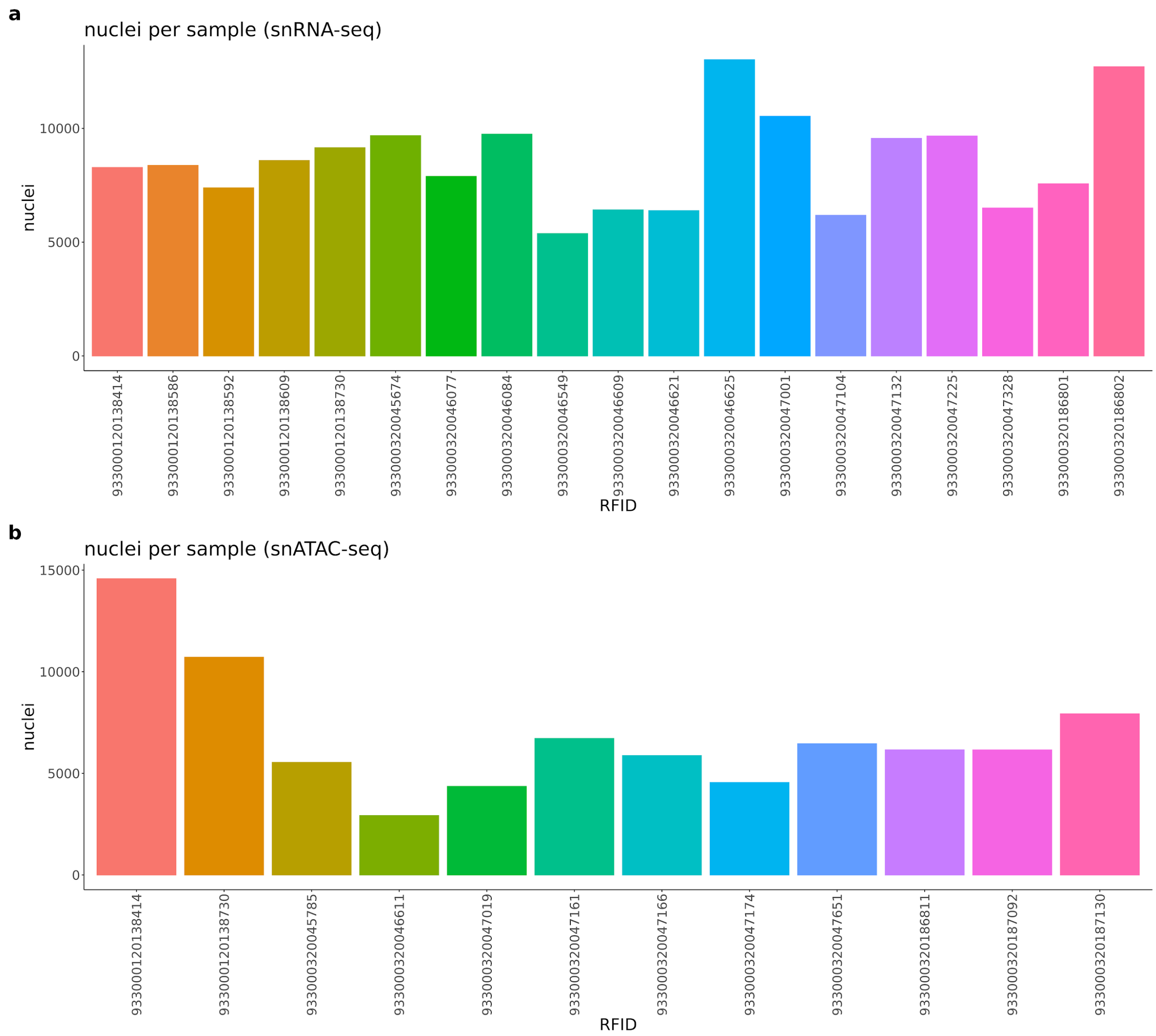

Supplementary Figure 7. Number of nuclei coming from each sample in the **a)** snRNA-seq and **b)** snATAC-seq datasets. Mean nuclei per sample is a) 8579 in the snRNA-seq, and b) 6826 in the snRNA-seq.

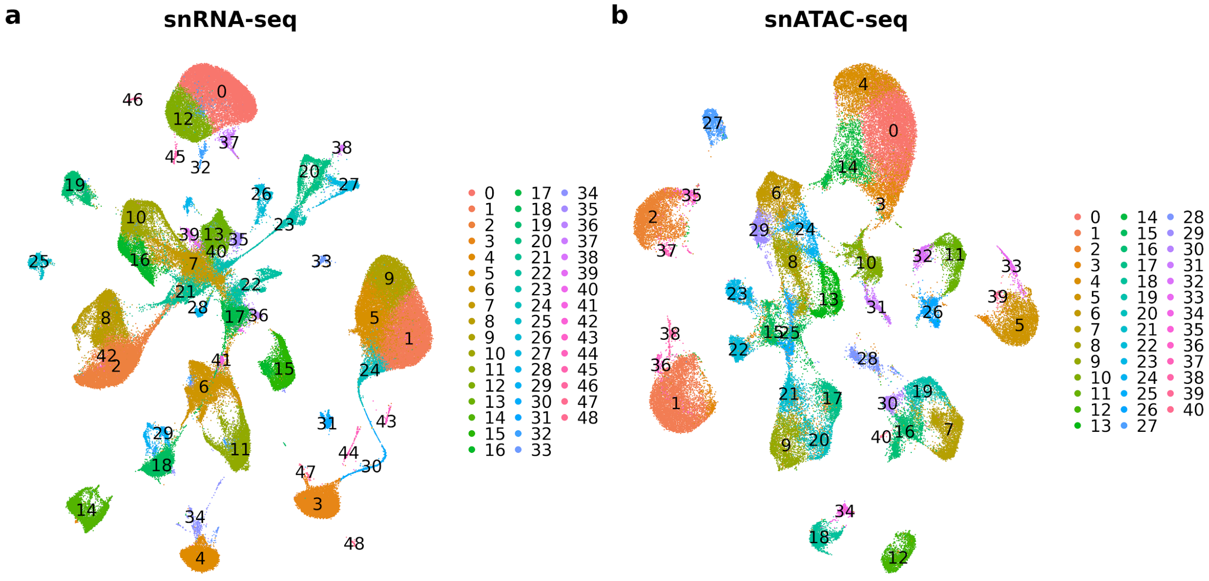

Supplementary Figure 8. UMAP visualization of the clusters identified in integrated single-cell data sets. **a)** Clustering of integrated snRNA-seq dataset revealed 49 clusters. We first performed a k-nearest neighbors analysis (KNN) using the first 30 dimensions calculated by reciprocal principal component analysis (PCA). This was implemented with the FindNeighbors() function in Seurat. Next we used a modularity optimization technique using the Louvain algorithm to cluster the data, implemented with the FindClusters() function in Seurat with a resolution parameter of 0.8. **b)** Clustering of integrated snATAC-seq data revealed 41 clusters. Latent semantic indexing (LSI) was used for dimensionality reduction rather than PCA. The first 30 dimensions minus the first dimension were used for KNN and clustering and the algorithm used for clustering was the smart local moving (SLM) algorithm. These steps were implemented with the same Seurat functions.

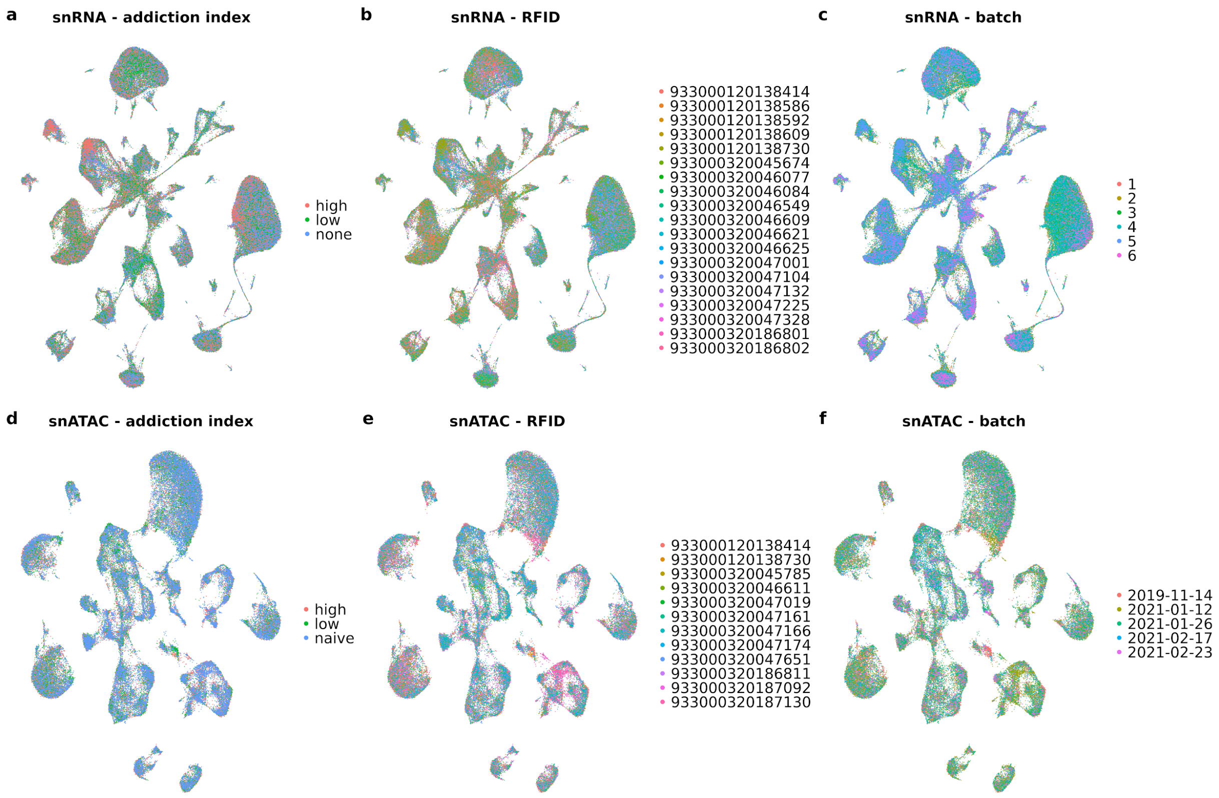

Supplementary Figure 9. UMAPS of snRNA-seq and snATAC-seq profiles, respectively, following batch correction of integrated datasets, grouped on: addiction index (a, d), rat sample (b, e), and batch information (c, f). These plots demonstrate that cells do not cluster by any of these covariates following batch correction. Integration and batch correction of the snRNA-seq dataset was performed using SCTransform while Harmony was used for the snATAC-seq dataset.

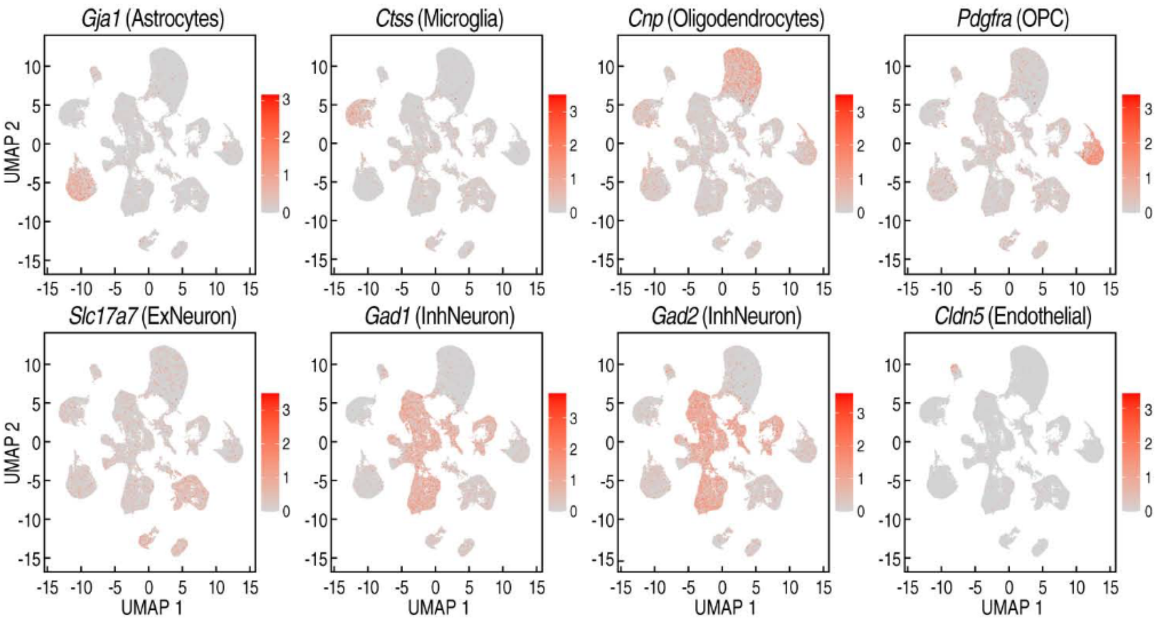

Supplementary Figure 10. Feature plots showing gene activity of marker genes for each major cell type in the snATAC-seq data. Gene activity was calculated with the `GeneActivity()` function in Signac. This quantifies the number of fragments mapping anywhere within a 2kb window of an annotated gene in the genome. The gene activity information was used for integration of the snATAC-seq dataset with the snRNA-seq dataset and for imputing gene expression into the cells of the snATAC-seq dataset (see Fig. 2d).

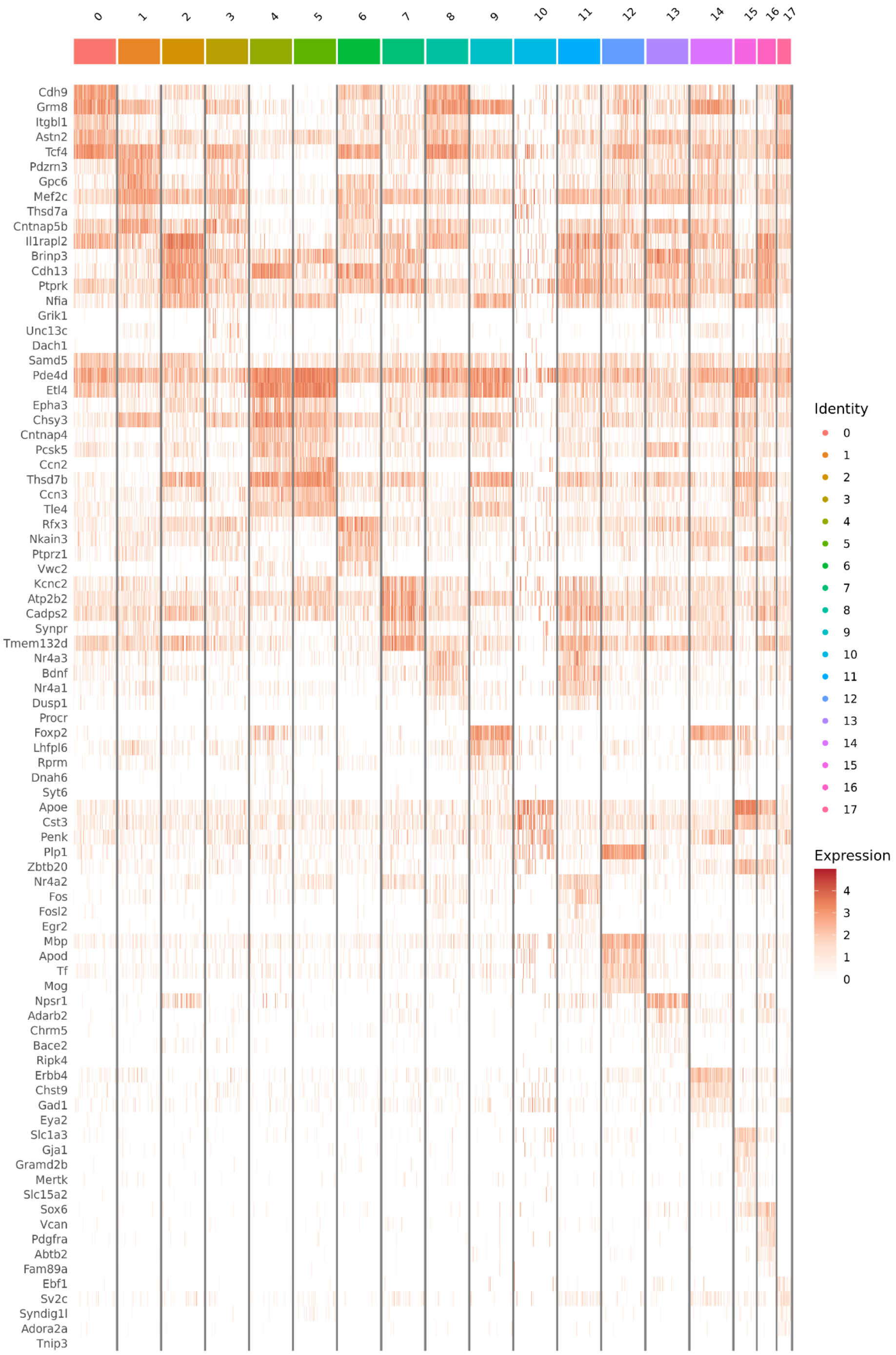

Supplementary Figure 11. Heatmap of top five marker gene expression within subclustered excitatory neurons.

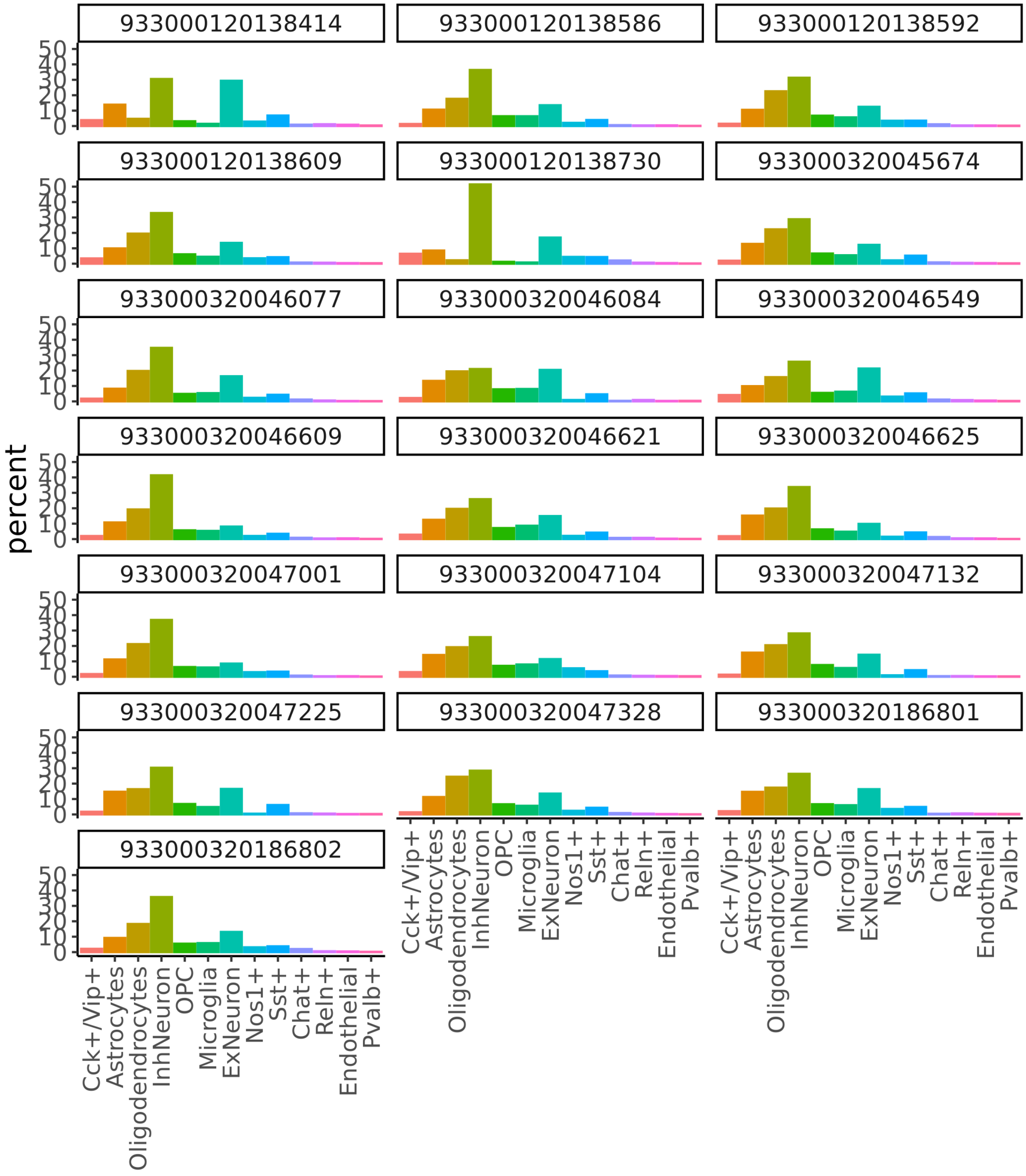

Supplementary Figure 12. Barplots show counts of each cell type within each sample, labeled by RFID, for the snRNA-seq samples.

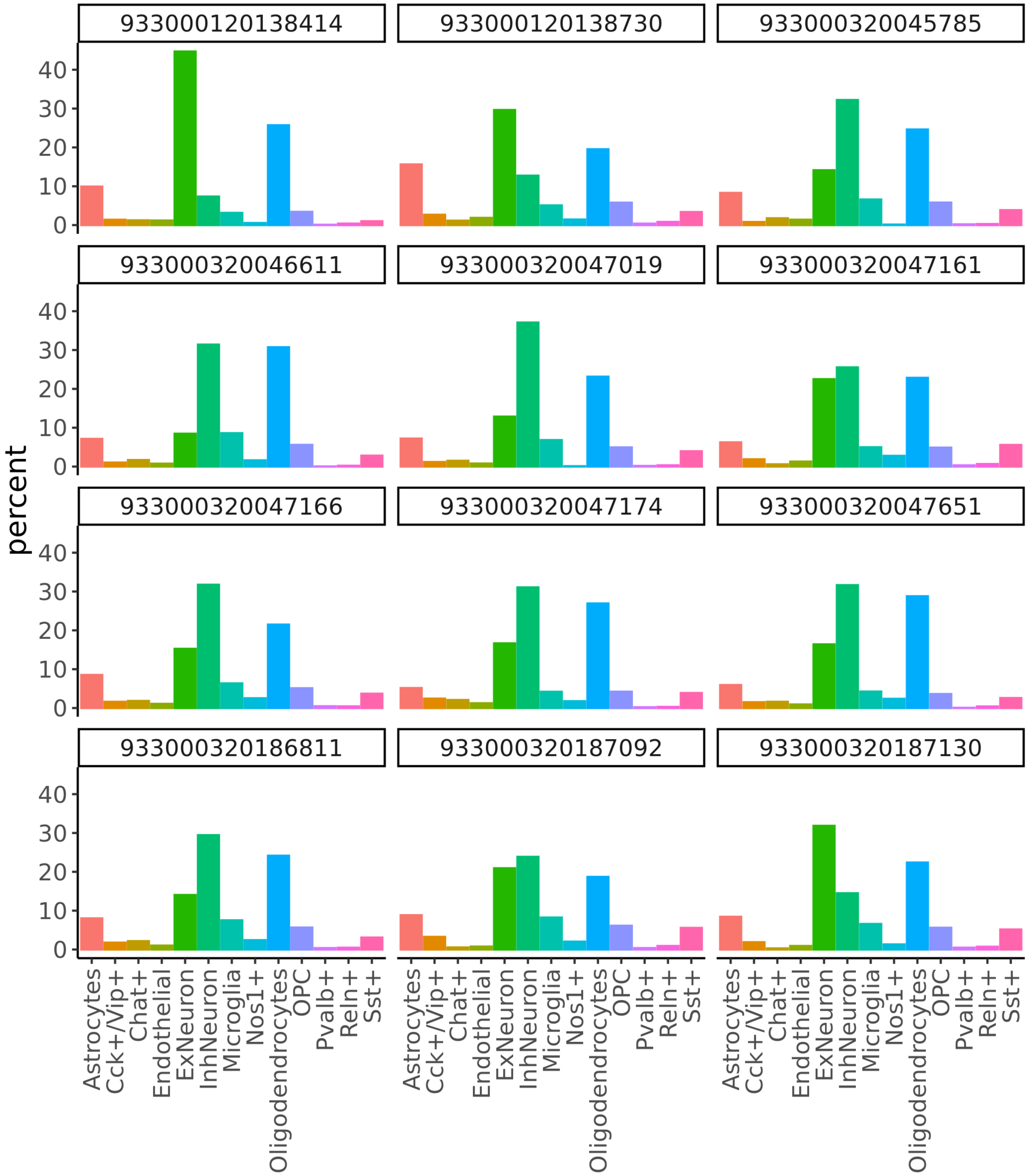

Supplementary Figure 13. Barplots show counts of each cell type within each sample, labeled by RFID, for the snATAC-seq samples.

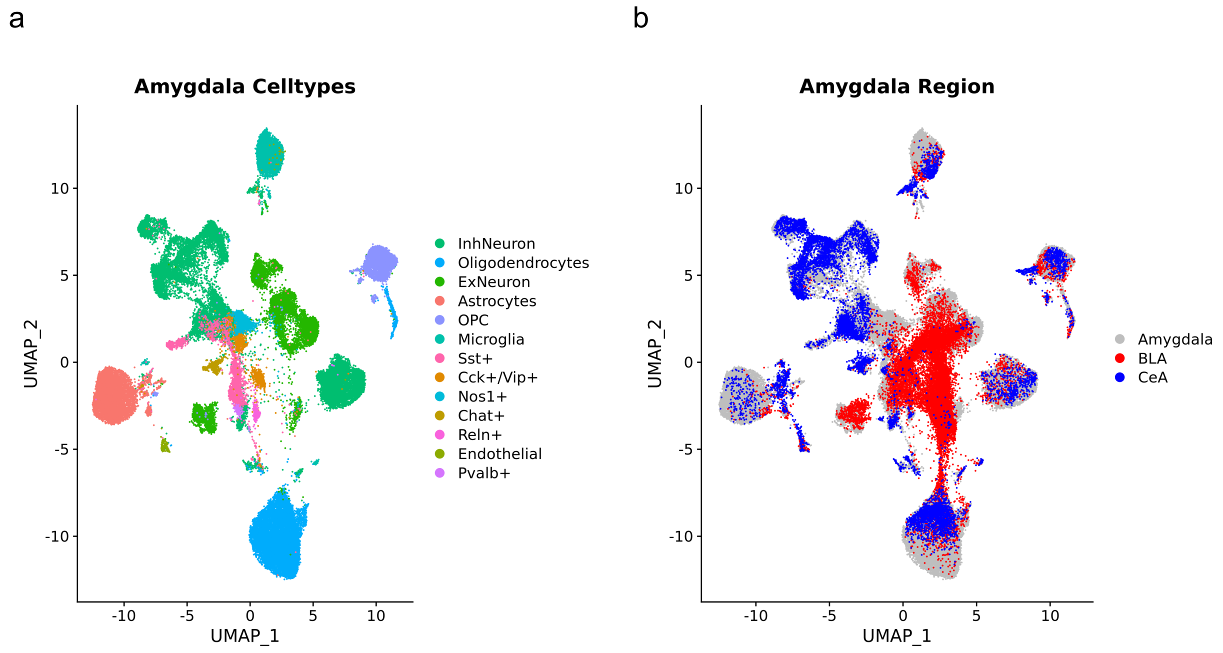

Supplementary Figure 14. Nearest neighbors co-clustering of snRNA-seq from a CEA sample, a BLA sample, and the whole amygdala samples from all of the naive rats in our study, visualized with UMAP. **a)** UMAP with cells colored by cell type cluster assignments. **b)** UMAP with cells colored by source tissue, where “Amygdala” refers to the set of all amygdala samples from the naive rats in our study.

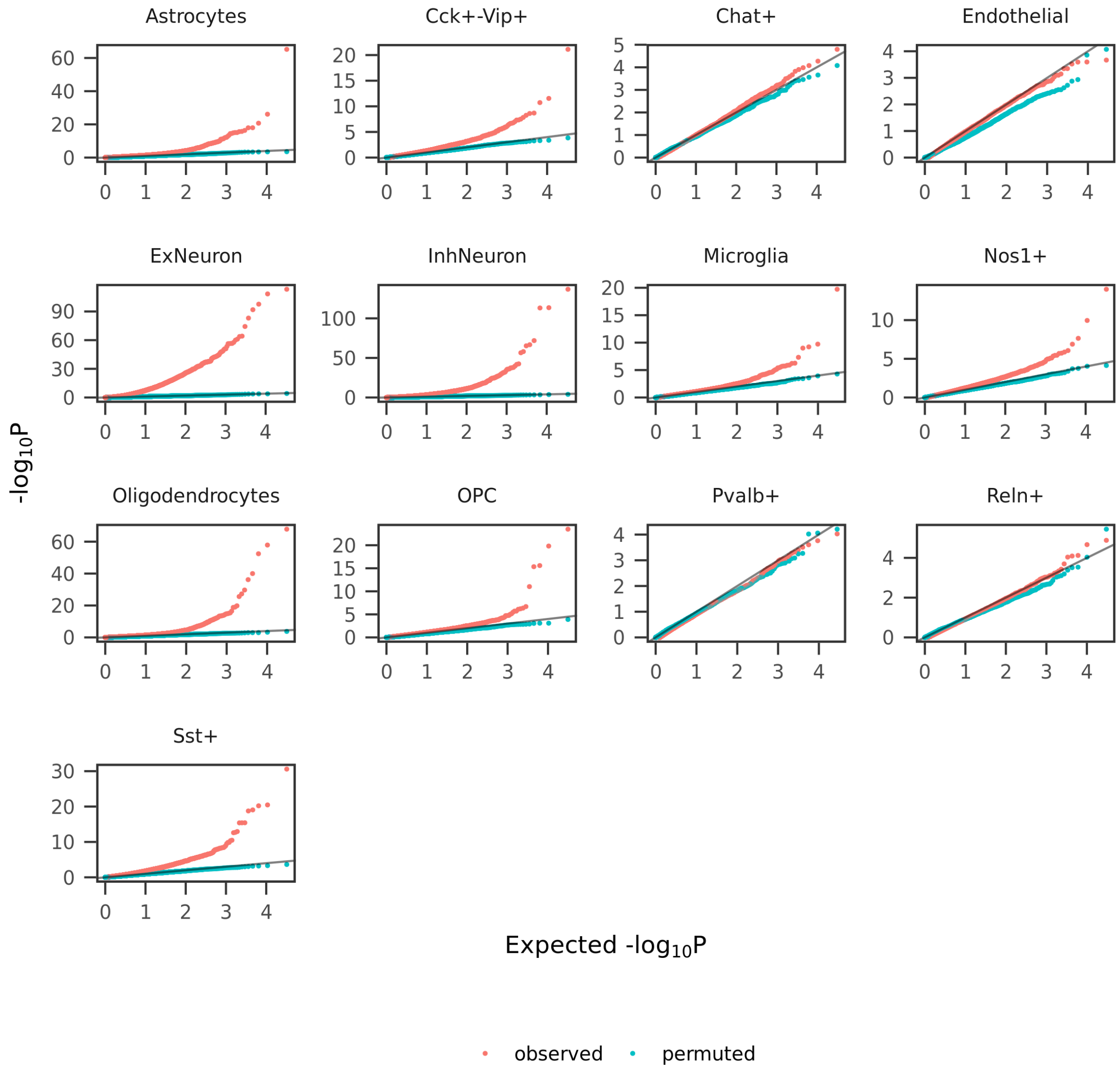

Supplementary Figure 15. QQ plots showing distribution of p-values for our differential gene expression analysis performed on our observed versus permuted data (AI labels associated with each cell were shuffled). The negative binomial test was the statistical test used for the analysis of both the observed and permuted datasets.

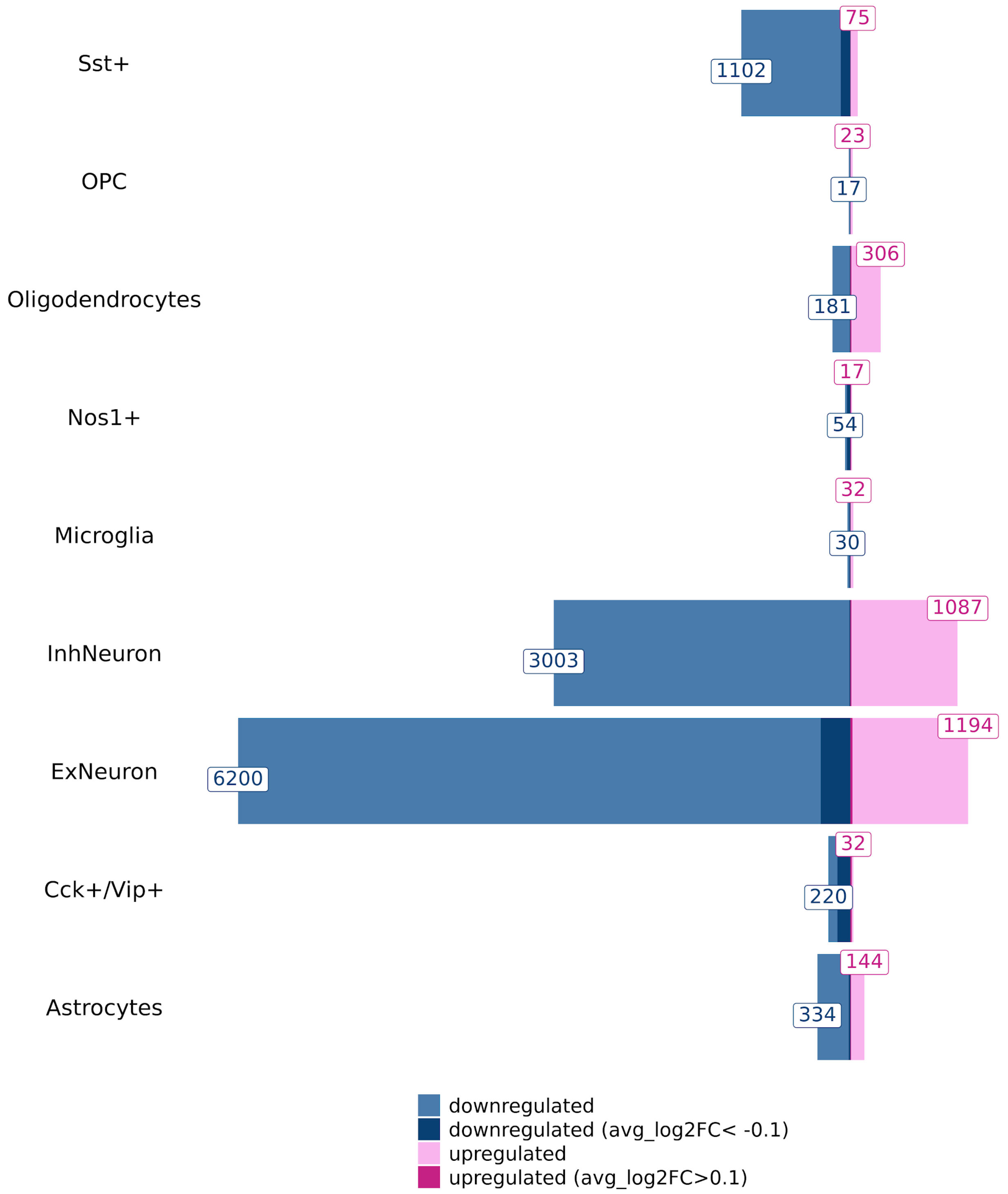

Supplementary Figure 16. Barplot showing numbers (labeled) of significant (FDR<10%) up- and downregulated DEGs by cell type. Darker shades indicate DEGs with a large fold change (abs(avg_$\log_2$FC)≥0.1).

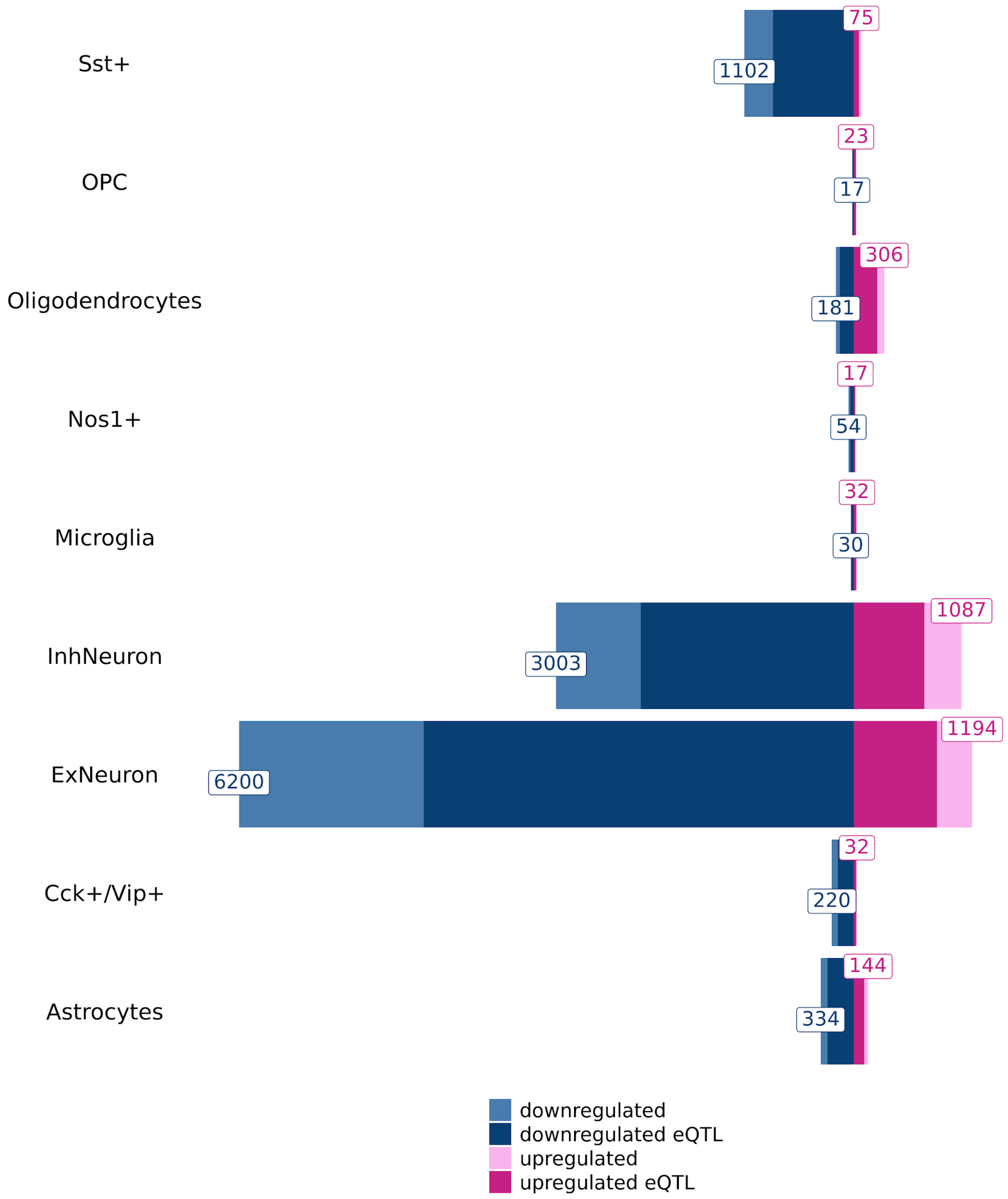

Supplementary Figure 17. Barplot showing numbers (labeled) of significant (FDR<10%) up- and downregulated DEGs by cell type. Darker shades indicate DEGs that are also significant eQTLs in rat brain tissues.

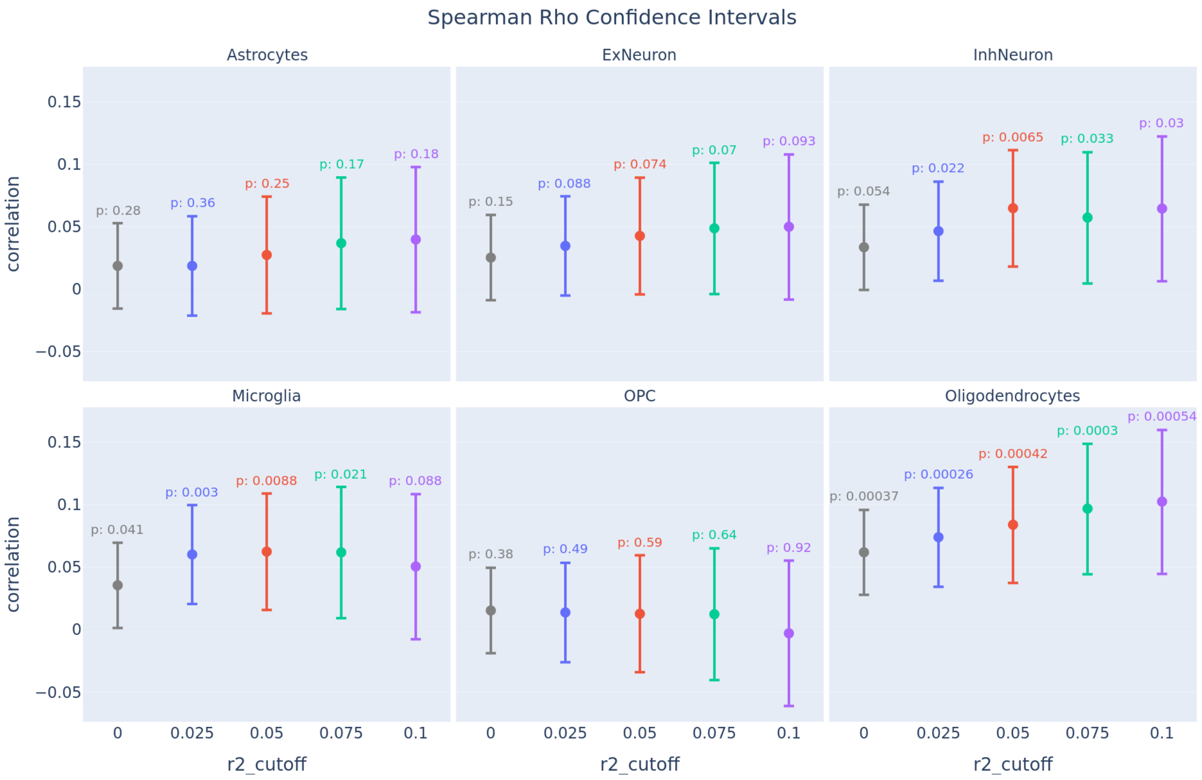

Supplementary Figure 18. Dot and whisker plots summarizing Spearman's correlation between predicted vs. observed differences in gene expression (high vs. low AI) for each major cell type (n=3,292 genes). We filtered the genes in the predicted set based on the r^2 metric, or predictive accuracy, of their predictive models. Each color corresponds to a different cutoff for r^2. We see significant correlations (p<0.05, by asymptotic t approximation and two sided Student's t-test) in microglia, oligodendrocytes and inhibitory neurons. We also observe a general trend of increasing correlation coefficients ρ as we increase the r^2 cutoff. Spearman's correlation coefficient ρ is plotted on the y-axis; r^2 cutoff is plotted on the x-axis. Spearman's ρ is denoted as the center dot, with whiskers indicating the 95% confidence interval.

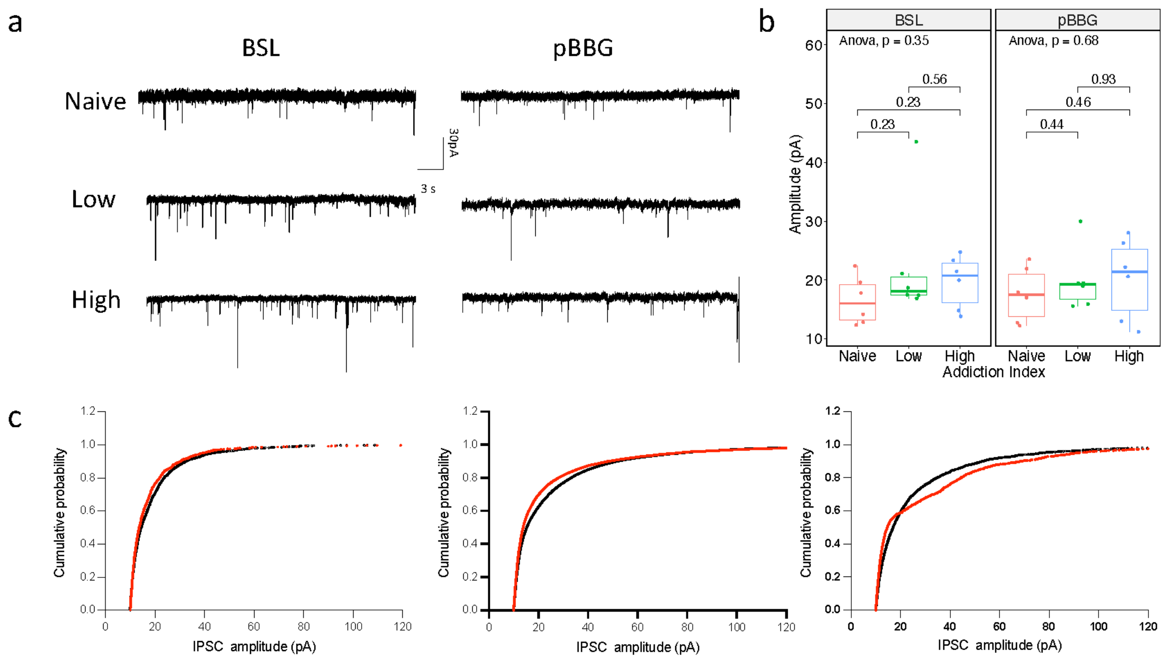

Supplementary Figure 19. Summary of electrophysiology experiments studying GABA transmission in the central amygdala. **a)** Representative traces of sIPSC frequencies for baseline (BSL) and following treatment with pBBG (pBBG) in naive, low AI and high AI rats. **b)** ANOVA F test comparing mean amplitude in BSL vs. pBBG across naive, low AI and high AI rats (degrees of freedom = 2, n = 6 for each AI group). Each point represents data for an individual rat. Boxplot hinges span the interquartile range (25th to 75th percentile), with the whiskers extending to the farthest point that is no more than 1.5 times the interquartile range from the hinge. Median is indicated by the center line. **c)** Cumulative probability plots of the peak amplitude for naive, low and high rats.

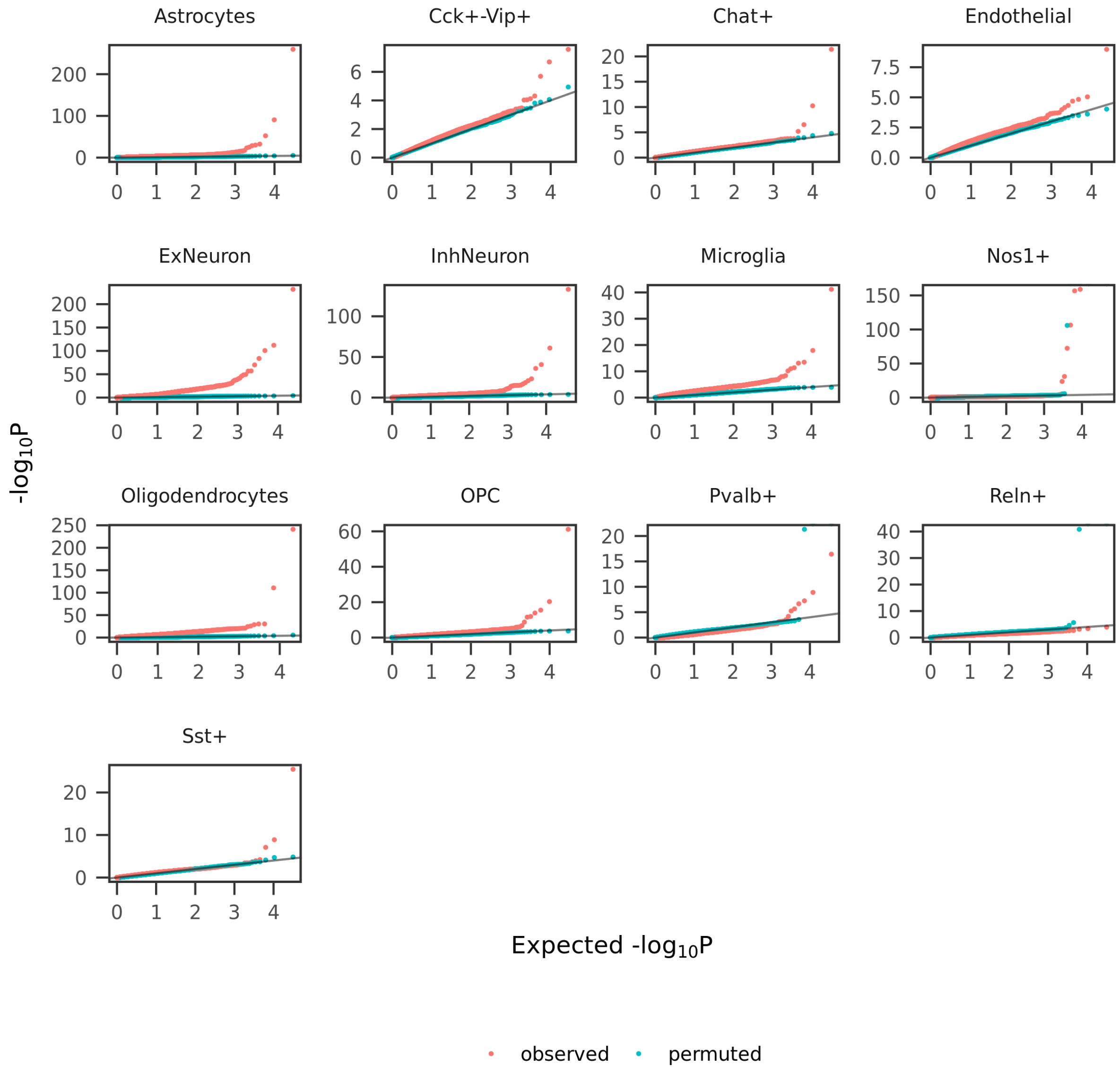

Supplementary Figure 20. QQ plots showing distribution of p-values for our differential peak accessibility analysis performed on our observed versus permuted data (AI labels associated with each cell were shuffled). A two-sided negative binomial test was used for the analysis of both the observed and permuted datasets.

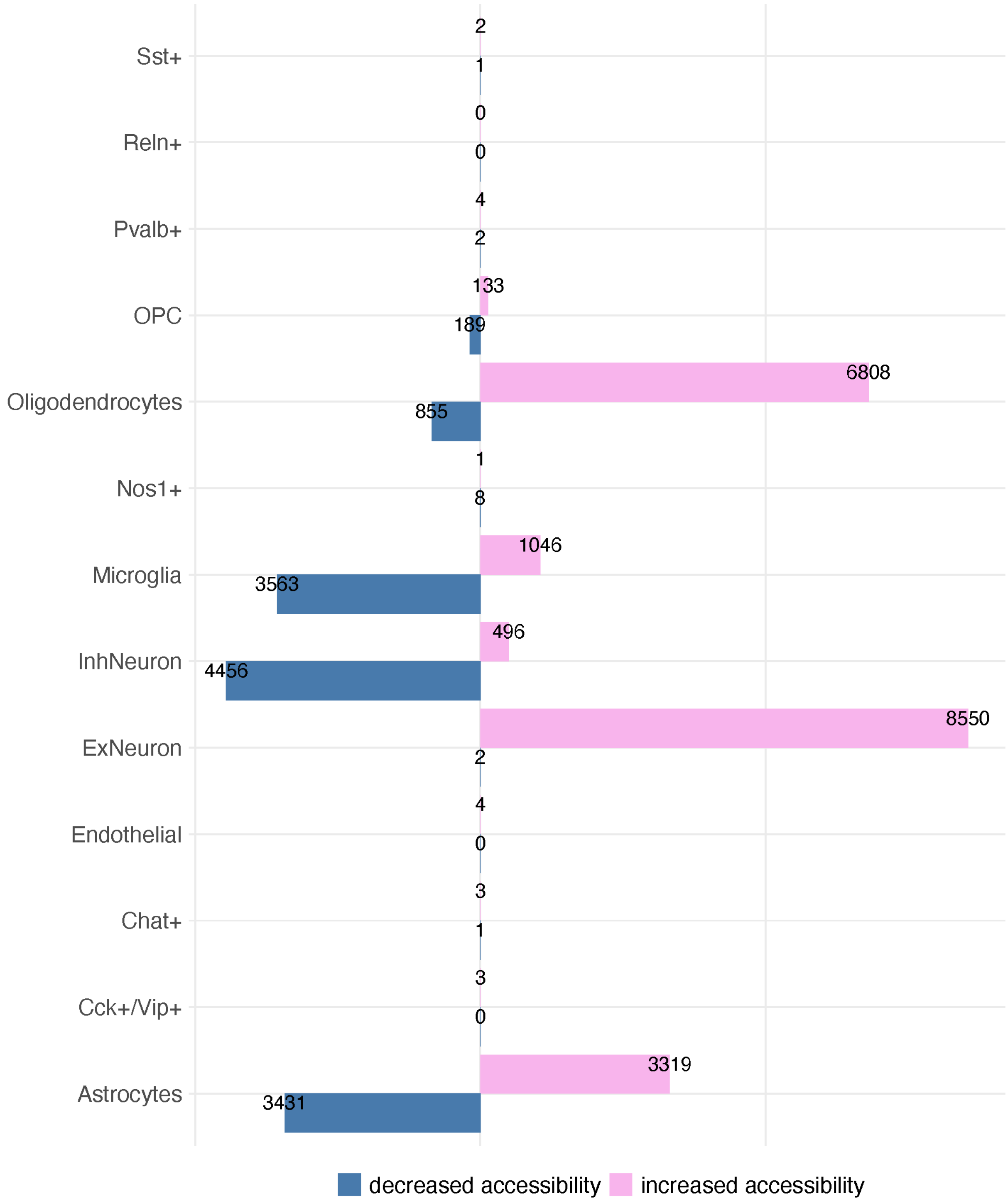

Supplementary Figure 21. Bar plot showing number of significant (FDR<10%) differentially accessible peaks between high vs. low rats in each cell type.

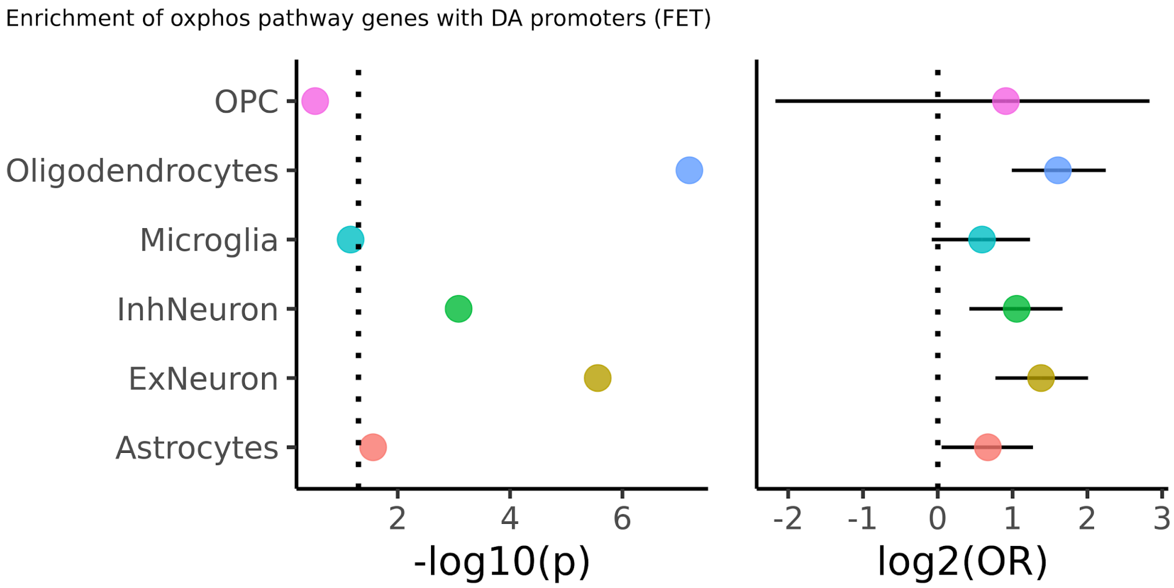

Supplementary Figure 22. Dot plots showing results of two-sided Fisher's Exact Test measuring enrichment of differentially accessible promoter regions (FDR<10%) in DEGs (FDR<10%) compared to non-DEGs for genes belonging to the oxidative phosphorylation pathway (as defined by the KEGG database). Data are presented as log2(OR) +/- 95% confidence interval. Statistics and sample sizes are provided in Supplementary Tables 4 and 5.

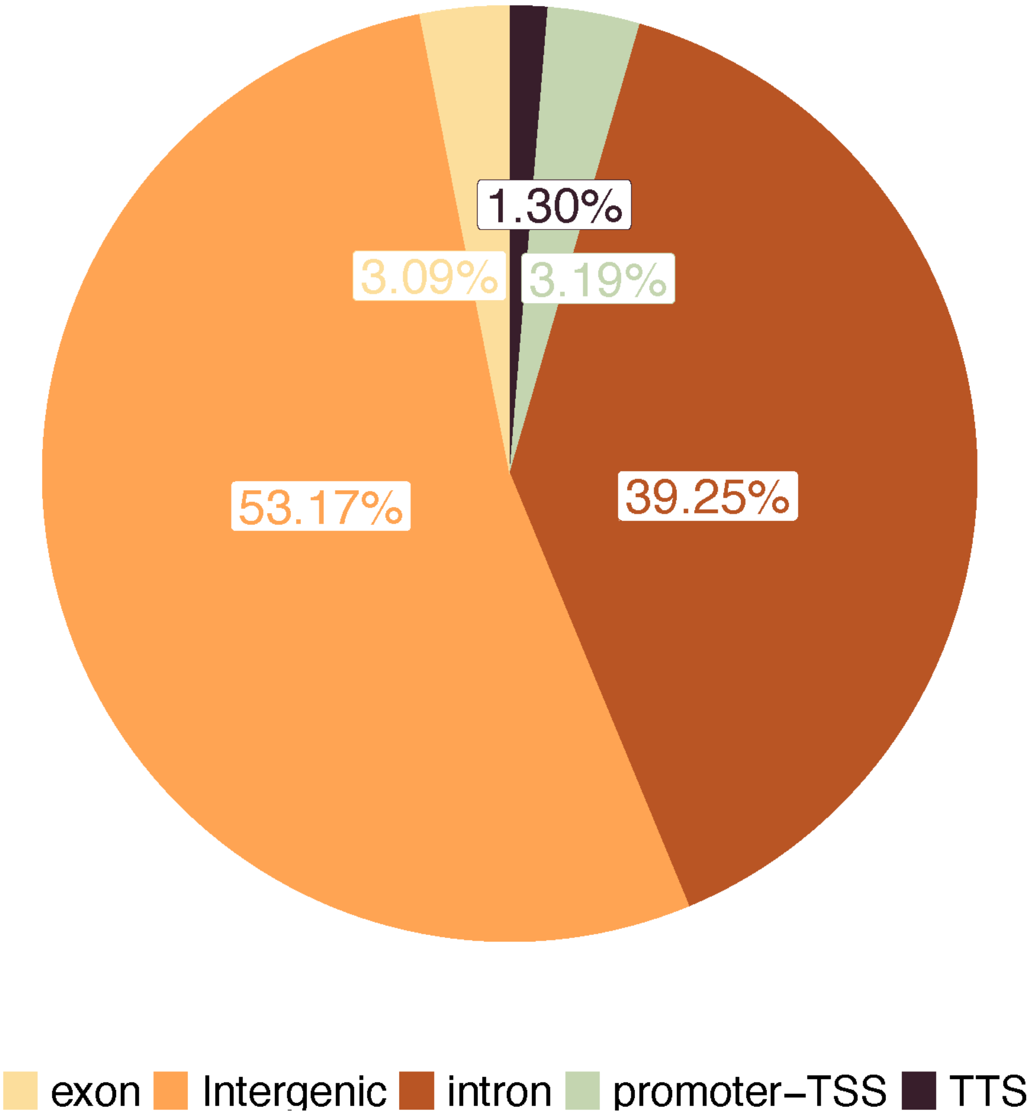

Supplementary Figure 23. Pie chart showing genomic annotations of all OCRs in our snATAC-seq dataset across all rats.

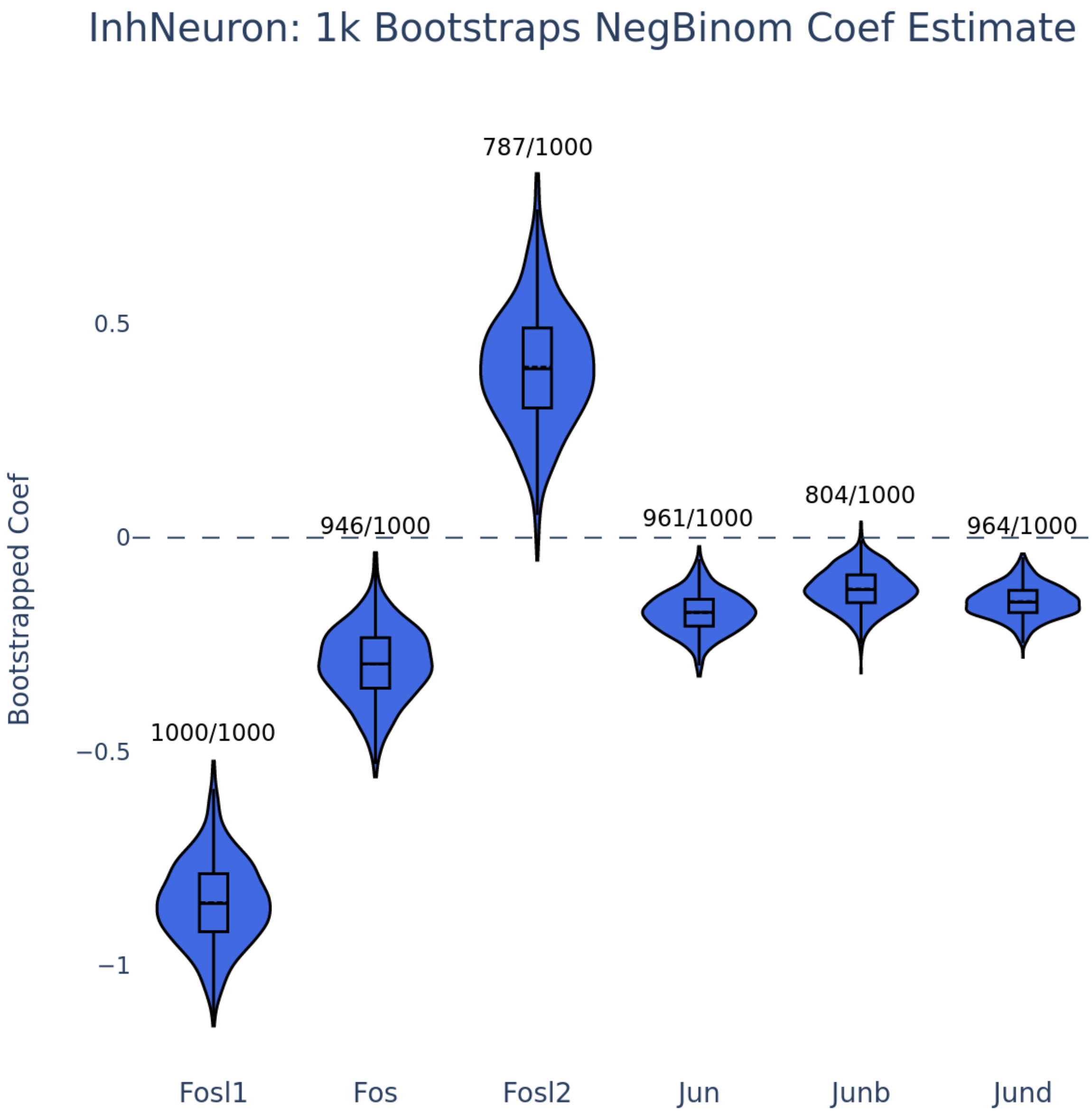

Supplementary Figure 24. Violin plots showing DEG analysis in InhNeurons over 1000 bootstrap iterations. Each violin shows the distribution of log2FC results per iteration. The fraction represents the number of significant iterations (FDR<10%). Boxplot hinges are the 25th and 75th percentiles; whiskers extend to the minimum and maximum; center line is the median; and dotted line is the mean.

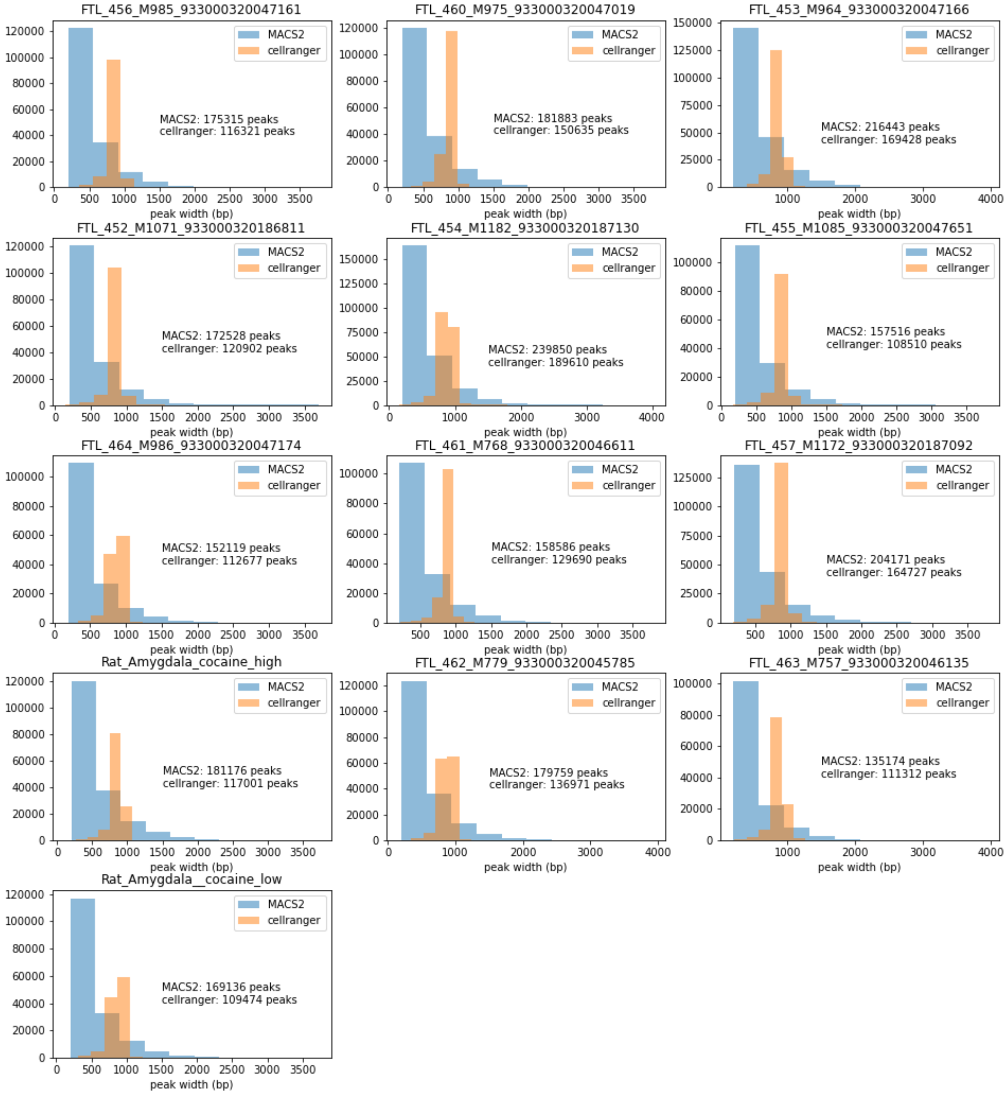

Supplementary Figure 25. Histograms showing distribution of peak sizes for peaks called by MACS2 (on the BAM files for the snATAC-seq data) versus CellRanger's internal peak calling algorithm. MACS2 calls smaller, more precise peaks.

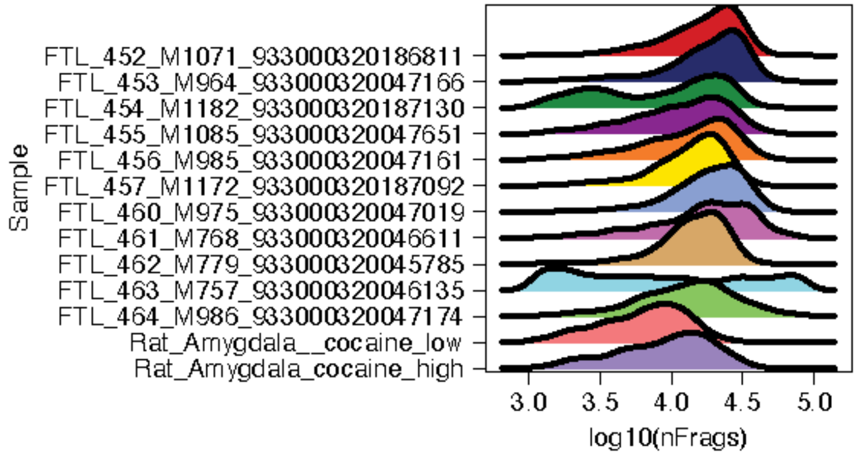

Supplementary Figure 26. Ridge plot quantifying the number of unique fragments (log10(nFrags)) per sample in the ATAC. Sample FTL_463_M757_933000320046135 was removed at this step and not included in any of our downstream snATAC-seq analyses due to its low number of fragments.

Supplementary Tables

Supplementary Table 1. Number and percentage of nuclei per cell type for snRNA-seq and snATAC-seq.

cluster	ncells.snRNA	percent.snRNA	ncells.snATAC	percent.snATAC
Astrocytes	19651	12.05560634	7337	8.957173552
Cck+/Vip+	3959	2.428789654	1496	1.82635023
Chat+	1628	0.9987546241	1043	1.273317707
Endothelial	699	0.4288264633	949	1.158560406
ExNeuron	23943	14.68868671	20169	24.6227659
InhNeuron	52579	32.25646154	18208	22.22873327
Microglia	8834	5.419532156	4574	5.58404141
Nos1+	4114	2.523879929	1232	1.50405313
Oligodendrocytes	29140	17.87697159	19391	23.67296611
OPC	9780	5.999889573	4031	4.921134876
Pvalb+	423	0.2595044263	213	0.2600351597
Reln+	1008	0.6183935265	426	0.5200703194
Sst+	7245	4.444703472	2843	3.470797929

Supplementary Table 2. Results of Chi-squared test with Yates' continuity correction for enrichment of significant DEGs (FDR<10%) that also have eQTLs in the rat brain in each cell type. The q-value column provides p-values that are adjusted for multiple testing using the Benjamini-Hochberg method.

statistic	p.value	parameter	celltype	q.value
32.00156145	1.54E-08	1	Astrocytes	9.24E-08
5.620791222	0.017748634	1	Cck+-Vip+	0.050195845
263.0825833	3.65E-59	1	ExNeuron	3.29E-58
115.3577062	6.57E-27	1	InhNeuron	5.26E-26
8.589987545	0.003380163	1	Microglia	0.013520653
0.293805179	0.587792337	1	Nos1+	0.587792337
30.98112175	2.61E-08	1	Oligodendrocytes	1.30E-07
5.724274495	0.016731948	1	OPC	0.050195845
43.28438553	4.73E-11	1	Sst+	3.31E-10

Supplementary Table 3. Spearman correlations between difference in mean predicted expression and observed avg_logFC of expression between high vs. low AI rats for subsets of genes passing each Pearson r^2 cutoffs for gene expression prediction models. P-values for Spearman correlations are computed using the cor.test function in R, which uses an asymptotic t approximation and Student's two-sided t-test. The p-values are not adjusted for multiple comparisons.

celltype	r2_cutoff	spearman_rho	pvalue	ci_low	ci_high	n_genes
Astrocytes	0	0.018701122	0.283414263	-0.015471691	0.052830298	3292
Astrocytes	0.025	0.018691166	0.357453182	-0.021121508	0.058444654	2426
Astrocytes	0.05	0.02746218	0.250335653	-0.019368071	0.074172197	1754
Astrocytes	0.075	0.036876648	0.170493112	-0.015866717	0.08941536	1383
Astrocytes	0.1	0.039827961	0.180354767	-0.018455348	0.097841507	1133
ExNeuron	0	0.025345141	0.145978786	-0.00882543	0.059456588	3292
ExNeuron	0.025	0.034691131	0.087575896	-0.00511289	0.074385397	2426
ExNeuron	0.05	0.042715472	0.073695635	-0.004098078	0.089342205	1754
ExNeuron	0.075	0.048777171	0.069770271	-0.003945496	0.101229416	1383
ExNeuron	0.1	0.049994674	0.092565284	-0.008269905	0.107920939	1133
InhNeuron	0	0.033607128	0.053848494	-0.000556439	0.067692338	3292
InhNeuron	0.025	0.046521106	0.021938511	0.006736615	0.086158555	2426
InhNeuron	0.05	0.064898807	0.006549113	0.018148579	0.111365876	1754
InhNeuron	0.075	0.057359357	0.03292901	0.004660927	0.109740074	1383
InhNeuron	0.1	0.064577247	0.0297394	0.00636067	0.122357559	1133
Microglia	0	0.035639761	0.040880673	0.001478633	0.069717804	3292
Microglia	0.025	0.060322746	0.002955403	0.020575177	0.099879931	2426
Microglia	0.05	0.062580891	0.008750828	0.015821879	0.109066777	1754
Microglia	0.075	0.061998527	0.021123002	0.009316451	0.114337383	1383
Microglia	0.1	0.05067189	0.08822582	-0.007591011	0.108591918	1133
OPC	0	0.015433019	0.376048454	-0.01873979	0.049569812	3292
OPC	0.025	0.01388068	0.494375796	-0.025930607	0.053648007	2426
OPC	0.05	0.012821107	0.59154584	-0.034004655	0.059590703	1754
OPC	0.075	0.012455566	0.64350377	-0.040283405	0.065125329	1383
OPC	0.1	-0.002824071	0.924351618	-0.061054529	0.055425544	1133

Oligodendrocytes	0	0.062043491	0.000368203	0.027939783	0.096002933	3292
Oligodendrocytes	0.025	0.074118064	0.000258399	0.034422637	0.113580003	2426
Oligodendrocytes	0.05	0.084101822	0.000421907	0.037443861	0.130393894	1754
Oligodendrocytes	0.075	0.096983393	0.000303911	0.044498356	0.148934526	1383
Oligodendrocytes	0.1	0.102632954	0.000540258	0.044659402	0.159917563	1133

Supplementary Table 4. Results of two-sided Fisher's exact test measuring enrichment of DEGs with differentially accessible promoters.

estimate	p.value	conf.low	conf.high	celltype	n
2.055111486	1.13E-11	1.666981196	2.529757319	Astrocytes	12081
2.562388137	5.90E-139	2.37680767	2.762935807	ExNeuron	12590
1.628121904	2.29E-23	1.479369058	1.791250157	InhNeuron	12679
1.656572395	0.09135641968	0.8587672432	3.05865139	Microglia	11232
1.878582849	4.58E-10	1.535304305	2.298289358	Oligodendrocytes	11886
3.196809822	0.2764188242	0.07779117094	19.528072	OPC	11646

Supplementary Table 5. Results of two-sided Fisher's exact test measuring enrichment of differentially accessible promoter regions (FDR<10%) in genes belonging to the oxidative phosphorylation pathway.

estimate	p.value	conf.low	conf.high	celltype	n
1.590213356	0.0274138768	1.036306859	2.4171411	Astrocytes	12081
0	1	0	Inf	Cck+-Vip+	11843
0	1	0	Inf	Chat+	11275
0	1	0	542.2633381	Endothelial	9803
2.603860013	2.75E-06	1.705313547	4.02696631	ExNeuron	12590
2.077830782	0.0008239595758	1.33980214	3.17681751	InhNeuron	12679
1.50676579	0.06905432995	0.9450140628	2.350921065	Microglia	11232
0	1	0	181.0640654	Nos1+	11672
3.044756686	6.43E-08	1.985216478	4.741095202	Oligodendrocytes	11886
1.879741784	0.2946158914	0.2220197518	7.117370798	OPC	11646
0	1	0	106.9419357	Pvalb+	9314
0	1	0	Inf	Sst+	12064

Supplementary Table 6. Results of two-sided Fisher's exact test measuring enrichment of differential peaks with TSS/promoter annotations.

estimate	p.value	conf.low	conf.high	celltype	q.value
18.81516404	0	17.77574265	19.9175995	Astrocytes	0
30.37291625	0.005840132	2.201548611	415.3908706	Endothelial	0.005840132
23.96758633	0	22.74043054	25.23646105	ExNeuron	0
17.57693406	0	16.48571015	18.74700536	InhNeuron	0
21.28725044	0	19.94126124	22.71783229	Microglia	0
27.72361083	0	26.31595904	29.21625885	Oligodendrocytes	0
10.28809974	7.95E-48	7.889775684	13.29876263	OPC	1.06E-47
30.37610297	0.000602346	4.068618798	226.6446137	Pvalb+	0.000688396
