# Supplementary material for: Single-nucleus genomics in outbred rats with divergent cocaine addiction-like behaviors reveals changes in amygdala GABAergic inhibition
Source: Nat Neurosci. 2023 Oct 5;26(11):1868–79. doi: 10.1038/s41593-023-01452-y (PMC10620093; doi:10.1038/s41593-023-01452-y)
Supplement: Supplementary file 2 — Reporting Summary [file 41593_2023_1452_MOESM2_ESM.pdf]

nature portfolio

Corresponding author(s): Francesca Telese
Graham McVicker

Last updated by author(s): 08-13-2023

Reporting Summary

Nature Portfolio wishes to improve the reproducibility of the work that we publish. This form provides structure for consistency and transparency in reporting. For further information on Nature Portfolio policies, see our Editorial Policies and the Editorial Policy Checklist.

Statistics

For all statistical analyses, confirm that the following items are present in the figure legend, table legend, main text, or Methods section.

n/a	Confirmed	
☐	☒	The exact sample size (*n*) for each experimental group/condition, given as a discrete number and unit of measurement
☐	☒	A statement on whether measurements were taken from distinct samples or whether the same sample was measured repeatedly
☐	☒	The statistical test(s) used AND whether they are one- or two-sided<br>*Only common tests should be described solely by name; describe more complex techniques in the Methods section.*
☐	☒	A description of all covariates tested
☐	☒	A description of any assumptions or corrections, such as tests of normality and adjustment for multiple comparisons
☐	☒	A full description of the statistical parameters including central tendency (e.g. means) or other basic estimates (e.g. regression coefficient) AND variation (e.g. standard deviation) or associated estimates of uncertainty (e.g. confidence intervals)
☐	☒	For null hypothesis testing, the test statistic (e.g. *F*, *t*, *r*) with confidence intervals, effect sizes, degrees of freedom and *P* value noted<br>*Give P values as exact values whenever suitable.*
☐	☒	For Bayesian analysis, information on the choice of priors and Markov chain Monte Carlo settings
☐	☒	For hierarchical and complex designs, identification of the appropriate level for tests and full reporting of outcomes
☐	☒	Estimates of effect sizes (e.g. Cohen's *d*, Pearson's *r*), indicating how they were calculated

Our web collection on statistics for biologists contains articles on many of the points above.

Software and code

Policy information about availability of computer code

Data collection	Operant chambers software MedPCIv v5, Sony Cell Sorter Software v2.1.2-5, Illumina NovaSeq instrument control software (sequencing)
Data analysis	All code used for data collection can be found on our GitHub repo: https://github.com/mcvickerlab/sn_cocaine_rats. Statistical analysis for the behavioral and electrophysiology data was done with Graphpad Prism v9.

For manuscripts utilizing custom algorithms or software that are central to the research but not yet described in published literature, software must be made available to editors and reviewers. We strongly encourage code deposition in a community repository (e.g. GitHub). See the Nature Portfolio guidelines for submitting code & software for further information.

Data

Policy information about availability of data

All manuscripts must include a data availability statement. This statement should provide the following information, where applicable:

- Accession codes, unique identifiers, or web links for publicly available datasets
- A description of any restrictions on data availability
- For clinical datasets or third party data, please ensure that the statement adheres to our policy

The datasets generated in the current study are available through the Gene Expression Omnibus (GSE212417).
The following publicly available datasets were used: Rattus norvegicus Ensembl v98 reference genome and genome assembly (Rnor_6.0, http://useast.ensembl.org/Rattus_norvegicus/Info/Index); JASPAR2022 transcription factor binding profiles for vertebrates (https://jaspar.genereg.net/); ENCODE Honeybadger 2 ChIP-seq

(https://personal.broadinstitute.org/meuleman/reg2map/); Liu et al. 201974 GWAS for tobacco and nicotine addiction summary statistics (https://www.ncbi.nlm.nih.gov/pmc/articles/PMC6358542/); RatGTEx Portal tissue-specific cis-eQTLs (https://ratgtex.org/download/); 1000 Genomes European reference panel (https://alkesgroup.broadinstitute.org/LDSCORE/); KEGG pathways (https://www.kegg.jp/kegg/rest/keggapi.html).
The HS rats genotype, predicted gene expression and behavioral data are available through the Zenodo repository https://doi.org/10.5281/zenodo.8242458

Research involving human participants, their data, or biological material

Policy information about studies with human participants or human data. See also policy information about sex, gender (identity/presentation), and sexual orientation and race, ethnicity and racism.

Reporting on sex and gender	N/A
Reporting on race, ethnicity, or other socially relevant groupings	N/A
Population characteristics	N/A
Recruitment	N/A
Ethics oversight	N/A

Note that full information on the approval of the study protocol must also be provided in the manuscript.

Field-specific reporting

Please select the one below that is the best fit for your research. If you are not sure, read the appropriate sections before making your selection.

☒ Life sciences ☐ Behavioural & social sciences ☐ Ecological, evolutionary & environmental sciences

For a reference copy of the document with all sections, see nature.com/documents/nr-reporting-summary-flat.pdf

Life sciences study design

All studies must disclose on these points even when the disclosure is negative.

Sample size	No sample size calculation was performed. We used all the samples available to us, and performed power analyses to determine our power for detecting differential features based on the number of samples that we had available and observed satisfactory results.
Data exclusions	Of the snATAC-seq samples available to us, we removed one rat from downstream analysis (RFID: 933000320046135) due to its low distribution of fragment sizes which was indicative of low quality.
Replication	Most steps in the analysis pipeline are reproducible; however, clustering cells has an element of stochasticity. This can be controlled for by using a random seed. We have provided our code on GitHub to ensure reproducibility.
Randomization	We used percent mitochondria reads and library prep date as covariates for differential gene expression analyses. We used number of peak region fragments, library batch date, and rat sample ID as covariates for differential chromatin accessibility analyses
Blinding	Experimenters were blinded to group allocation during behavioral data collection prior to brain collection and during brain processing for sequencing experiments. Investigators were not blinded to the samples being investigated after allocation of specimens to different behavioral groups because downstream analysis required comparisons between high and low groups.

Reporting for specific materials, systems and methods

We require information from authors about some types of materials, experimental systems and methods used in many studies. Here, indicate whether each material, system or method listed is relevant to your study. If you are not sure if a list item applies to your research, read the appropriate section before selecting a response.

Materials & experimental systems

n/a	Involved in the study
☒	☐ Antibodies
☒	☐ Eukaryotic cell lines
☒	☐ Palaeontology and archaeology
☐	☒ Animals and other organisms
☒	☐ Clinical data
☒	☐ Dual use research of concern
☒	☐ Plants

Methods

n/a	Involved in the study
☒	☐ ChIP-seq
☐	☒ Flow cytometry
☒	☐ MRI-based neuroimaging

Animals and other research organisms

Policy information about studies involving animals; ARRIVE guidelines recommended for reporting animal research, and Sex and Gender in Research

Laboratory animals	N/NIH heterogeneous stock (HS) rat (RRID:RGD_2314009) and ACI/EurMcw rats (RRID:RRRC_00284). Rats were 3-4 weeks of age at the start of the experiments.
Wild animals	No wild animals were used in this study.
Reporting on sex	We used 57 HS rats for the behavioral experiments, of which 31 male rats were used for the generation of snRNA-seq and snATAC-seq data and 26 rats (13 female, 13 male) were used for cue-induced reinstatement. Specifically, for snRNA-seq we used 19 male rats (6 high AI, 6 low AI, 7 naive); for the snATAC-seq we used 12 male rats (4 high AI, 4 low AI, 4 naive). In addition, we used 15 female and male rats (5 high AI, 5 low AI, 5 naive) for the electrophysiology experiments.
Field-collected samples	No filed-collected samples were used in this study.
Ethics oversight	All protocols were reviewed and approved by the institutional Animal Care and Use Committee at the University of California San Diego.

Note that full information on the approval of the study protocol must also be provided in the manuscript.

Flow Cytometry

Plots

Confirm that:

☒ The axis labels state the marker and fluorochrome used (e.g. CD4-FITC).

☒ The axis scales are clearly visible. Include numbers along axes only for bottom left plot of group (a 'group' is an analysis of identical markers).

☒ All plots are contour plots with outliers or pseudocolor plots.

☒ A numerical value for number of cells or percentage (with statistics) is provided.

Methodology

Sample preparation	Nuclei were stained with DRAQ7 (#7406, Cell Signaling)
Instrument	Sony SH800
Software	SH800S software
Cell population abundance	NA
Gating strategy	The FACS gating strategy sorted based on particle size and DRAQ7 fluorescence.

☐ Tick this box to confirm that a figure exemplifying the gating strategy is provided in the Supplementary Information.
